# Implementing school malaria surveys in Kenya: towards a national surveillance system

**DOI:** 10.1186/1475-2875-9-306

**Published:** 2010-10-30

**Authors:** Caroline W Gitonga, Peris N Karanja, Jimmy Kihara, Mariam Mwanje, Elizabeth Juma, Robert W Snow, Abdisalan M Noor, Simon Brooker

**Affiliations:** 1 Malaria Public Health & Epidemiology Group, Kenya Medical Research Institute-Wellcome Trust Research Programme, Nairobi, Kenya; 2 Division of Vector Borne and Neglected Tropical Diseases, Ministry of Public Health & Sanitation, Nairobi, Kenya; 3 Eastern and Southern Africa Centre of International Parasite Control, Kenya Medical Research Institute, Nairobi, Kenya; 4 Division of Malaria Control, Ministry of Public Health & Sanitation, Nairobi, Kenya; 5 Centre for Tropical Medicine, Nuffield Department of Clinical Medicine, University of Oxford, CCVTM, Oxford, UK; 6 Faculty of Infectious and Tropical Diseases, London School of Hygiene and Tropical Medicine, UK

## Abstract

**Objective:**

To design and implement surveys of malaria infection and coverage of malaria control interventions among school children in Kenya in order to contribute towards a nationwide assessment of malaria.

**Methods:**

The country was stratified into distinct malaria transmission zones based on a malaria risk map and 480 schools were visited between October 2008 and March 2010. Surveys were conducted in two phases: an initial opportunistic phase whereby schools were selected for other research purposes; and a second phase whereby schools were purposively selected to provide adequate spatial representation across the country. Consent for participation was based on passive, opt-out consent rather than written, opt-in consent because of the routine, low-risk nature of the survey. All children were diagnosed for *Plasmodium *infection using rapid diagnostic tests, assessed for anaemia and were interviewed about mosquito net usage, recent history of illness, and socio-economic and household indicators. Children's responses were entered electronically in the school and data transmitted nightly to Nairobi using a mobile phone modem connection. RDT positive results were corrected by microscopy and all results were adjusted for clustering using random effect regression modelling.

**Results:**

49,975 children in 480 schools were sampled, at an estimated cost of US$ 1,116 per school. The overall prevalence of malaria and anaemia was 4.3% and 14.1%, respectively, and 19.0% of children reported using an insecticide-treated net (ITN). The prevalence of infection showed marked variation across the country, with prevalence being highest in Western and Nyanza provinces, and lowest in Central, North Eastern and Eastern provinces. Nationally, 2.3% of schools had reported ITN use >60%, and low reported ITN use was a particular problem in Western and Nyanza provinces. Few schools reported having malaria health education materials or ongoing malaria control activities.

**Conclusion:**

School malaria surveys provide a rapid, cheap and sustainable approach to malaria surveillance which can complement household surveys, and in Kenya, show that large areas of the country do not merit any direct school-based control, but school-based interventions, coupled with strengthened community-based strategies, are warranted in western and coastal Kenya. The results also provide detailed baseline data to inform evaluation of school-based malaria control in Kenya.

## Background

The epidemiology of malaria in sub-Saharan Africa (SSA) is in transition, with funding agencies dedicating substantial resources in tackling malaria and national governments making great efforts in increasing access to malaria control interventions. It is essential that this transition is accurately monitored in order to evaluate the impact of interventions but also to allow for better targeting of interventions. A number of studies provide evidence of declining malaria-related mortality and morbidity [1-6], but there is, surprisingly, little evidence of the impact of control on malaria transmission. This is most commonly measured on the basis of the parasite rate (PR), since it is readily measured in the field and provides reliable information on other measures of malaria transmission, including the entomological inoculation rate and basic reproductive number [7]. Consequently, estimates of PR form the best evidence base for planning, implementing and evaluating control, with PR among children aged two to 10 years providing a standard measure of PR [8]. To date, malaria monitoring and evaluation of interventions in malaria endemic countries in SSA has been mainly based on periodic national household surveys, including malaria indicator survey [9] as well as malaria modules of demographic health surveys [10] and multiple indicator cluster surveys [11], where young children and pregnant women form the sample population. The principal advantages of such household surveys are that they adequately capture the underlying variation in the sampled population and the flexibility of data collection instruments which can accommodate a number of questions on a variety of topics. However, household surveys are expensive, time consuming and labour intensive, and generally only undertaken every 3-5 years and therefore not ideal for routine monitoring at local levels. Furthermore, estimates of *Plasmodium *infection collected among young children and pregnant may not be optimal due to the modifying presence of maternal antibodies and sequestered parasites [12]. A cheaper and rapid complementary approach to household surveys would be to use the existing school system for school-based malariometric surveys [12].

Historically, such school surveys were routinely conducted as part of malaria surveillance in Africa [12] and today, school surveys for helminth infections are an essential component of the design and evaluation of helminth control [13,14]. Learning from these historic and contemporary experiences, this paper reports the study design and main findings of a series of large-scale school malaria surveys in Kenya, with a view to informing future nationwide school-based surveillance. Particular guidance is provided on the consent for participation, field logistics and implementation of the survey and reflection is made on the ethical, practical and methodological issues encountered in conducting malaria surveys in schools.

## Methods

### The Kenyan context

The epidemiology of malaria in Kenya has been changing with reported reductions in malaria associated hospital admissions and mortality in children under the age of five years [15-17]. These changes have been, in part, attributed to the increase in coverage and access to malaria control interventions, such as insecticide-treated nets (ITNs), artemisinin-based combination therapy (ACT) and indoor residual spraying (IRS) [18]. In an effort to scale up ITN coverage, Kenya has adopted several ITN distribution strategies over the years, including social marketing, subsidized nets through the maternal and child clinics, and mass campaigns [18,19]. Other malaria control efforts include the change of the treatment policy in 2004 and implemented in 2006 to adopt the more efficacious ACT as well as IRS in the epidemic prone districts.

In 2009, the Government of Kenya launched its National Malaria Strategy (NMS), 2009-2017. This identified the need to tailor malaria control interventions to the local diversity of malaria risk, target specific population sub-groups to achieve effective and sustainable control, and strengthen the surveillance, monitoring and evaluation systems [20]. One approach to target population sub-groups includes the control of malaria in schools under a Malaria-free Schools Initiative. These plans for school-based malaria control build on recent success in delivering deworming through schools in Kenya. Implementation of the national programme was guided by school surveys of helminth infection which showed that mass treatment was only warranted in selected regions of the country [21] thereby increasing the efficiency of the programme. Before appropriate suites of malaria intervention can be planned efficiently for the Malaria-free schools initiative, equivalent data are required concerning the prevalence and distribution of malaria, anaemia, and intervention coverage across the country.

The Kenya NMS also included the proposal to undertake school malaria surveys to monitor trends of malaria transmission in the context of increasing intervention coverage. Such school surveys have a historical precedent in Kenya, dating back to the 1950 s, when the Division of Vector Borne Diseases (DVBD) was established and school surveys of malaria, helminths and other parasites were one of its core activities. Routine school survey stopped in the 1980 s due to financial constraints and deteriorating school enrolment rates [22].

The renewed potential for school malaria surveys builds on the increased funding for malaria surveillance but also recent improvements in primary school enrolment in Kenya. There are a total of 19,177 government primary schools, the majority (98.5%) of which are day schools with pupils living at home. Primary education in Kenya begins at the age of 6 or 7 years old after completion of a year of nursery school and includes eight years of schooling. The Kenyan school year runs from January to December. In the 1980 s and 1990 s, there was a growth of privately owned schools while the government schools deteriorated. In 2003, the Government of Kenya re-introduced free primary education, resulting in a marked increase in school enrolment. However, parents must pay fees for uniforms and other items and some poorer children still do not attend primary school. The overall net enrolment rate (NER: ratio of children of official school age who are enrolled in school to the population of the corresponding official school age.) in Kenya was 91.6% in 2007, but this ranged from 27.5% in North Eastern Province to 97.8% in Nyanza Province [23].

### Sample design and study population

**Figure 1:**
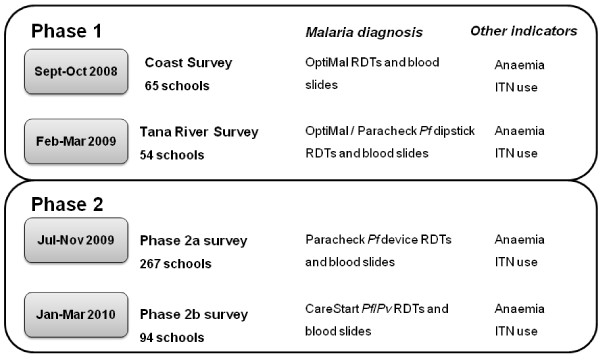
**Flow chart showing the two principle phases of the school malaria surveys, including timelines, rapid diagnostic test type and other indication data collected**.

**Figure 2:**
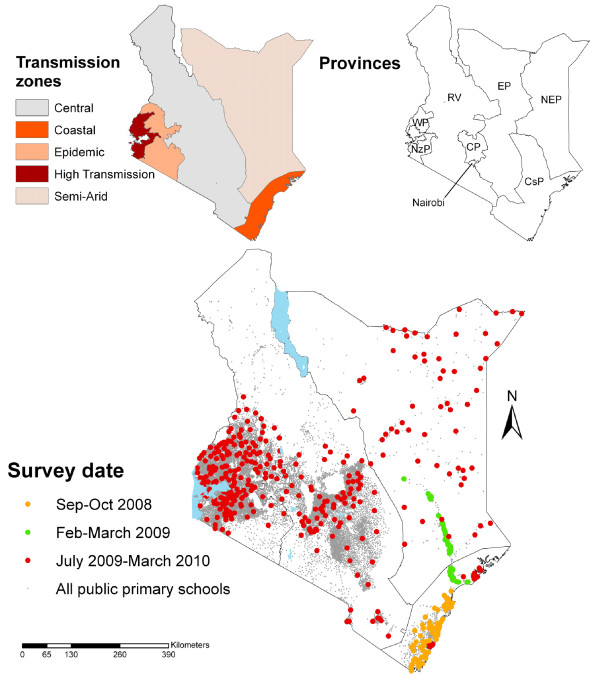
**The geographical distribution of the 480 sampled schools according to study phase**. These schools are overlaid on the distribution of all the 19,177 government, mixed primary schools in Kenya (Kenya Ministry of Education, 2008). Insert: Malaria transmission zones in Kenya based on a geostatistical model of *Plasmodium *prevalence [33] and the different level 1 administrative regions (Provinces: NzP = Nyanza Province, WP = Western Province; RV = Rift Valley Province; EP = Eastern Province; NEP = North Eastern Province; CP = Central Province; CsP = Coast Province).

The surveys were conducted in two principal phases (see Figures 1 and 2), based on the availability of resources at the time and intended purposes of each phase. The first phase was opportunistic in terms of malaria surveillance and included 65 schools sampled in three contiguous districts (the 1999 districts of Kwale, Kilifi and Malindi) along the Kenyan Coast, September-October 2008, as part of baseline surveys aimed at informing the implementation of the national school deworming programme (Figure 2). These surveys sought to define the prevalence of *Plasmodium *infection in a given district based on 95% confidence limits, 80% power, and a design effect of 2. Based on these assumptions, a minimum sample size of 16 schools per district was calculated as necessary to estimate prevalence of 5%, with 1% precision. An additional 54 schools were sampled as part of an evaluation of school net distribution programmes along the Tana River (Figure 2). These surveys meant that all districts in Coast Province, except Lamu District, were included in the first phase of the survey.

Based on these initial surveys, the second phase sought to create a nationwide sample of schools to allow for adequate spatial representation of malaria across the country, rather than provide precise estimation of prevalence at national and sub-national levels. Schools were selected from all remaining districts across the country with the exception of semi-arid districts in northern and southern Rift Valley Province (Figure 2). The sampling frame for this selection was the national schools census undertaken by the Ministry of Education (MoE) in 2008 of primary, secondary, public and private schools nationwide (MoE, 2008). For the purposes of the present survey, only public, mixed primary schools were selected as the universe of sampling, totalling 19,177. From this universe, approximately five schools in each of 70 district boundaries used during the 1999 census were selected. The selection of schools in each district was not weighted by population or fully random since schools were selected to provide adequate spatial spread of school locations, a requirement of geostatistical modelling of risk across space and time [24]. Finally, two over-sampling adjustments were undertaken: schools were over-sampled, disproportionate to district weighted school distributions, in the sparsely populated areas of North Eastern Province to increase the power of spatial interpolation of risk in these areas; and second, schools were purposively over-sampled schools in Central Kisii, Gucha and Rachuonyo districts where indoor residual spraying programmes were rolled out in 2008 to investigate impacts with time in these areas. A total of 361 schools were surveyed in the second phase during the second and third term of the 2009-2010 school year (June-November, 2009) and the first term (January-March, 2010) (Figure 2). The final sample included 480 schools sampled for malaria infection prevalence between September 2008 and March 2010.

Taking into account a combination of sample precision, logistics and costs, it was decided that a randomly selected sample of 100 children (plus 10 reserves) per school would be optimal as this was the number of children, which could practically be sampled in a single day. In each school, 11 boys and 11 girls were selected from each of classes 2-6 using computer generated random table numbers. If there were insufficient pupils in these classes, additional pupils were sampled from class 1. Some of the schools visited were small, and this meant that in these schools all children were selected to achieve the target sample size and fewer than 110 children were present and, therefore, examined.

### Team composition and logistics

Mobile survey teams consisted of a team leader, three laboratory technicians and three interviewers. Technicians were typically from the Division of Vector Borne and Neglected Tropical Diseases (DVBNTD) of Ministry of Public Health and Sanitation, holding diplomas or first degrees and who had extensive experience of conducting school surveys. Interviewers were either from the Ministry of Public Health and Sanitation or Ministry of Education, who had previous survey experience. Each team was supervised from an experienced researcher from the Kenyan Medical Research Institute (KEMRI) or KEMRI-Wellcome Trust Research Programme. These teams were accompanied by an education officer from the district education office who helped teams locate schools.

All team members underwent training in all survey procedures and received a field manual outlining the survey purpose and methods (see Additional file 1). Data collection occurred during the course of a school term, with each team travelling in a single vehicle with supplies necessary for a single term. An exception was heat sensitive supplies, such as malaria rapid diagnostic tests (RDTs) and haemoglobin microcuvettes, which were sent to teams on a weekly or fortnightly basis. Teams sent back blood slides and filter papers to Nairobi weekly in appropriate storage.

### Community sensitization

This took place at national, provincial and district levels before visiting the schools, using a cascade approach. At the national levels, the study was approved by the Division of Malaria Control, Ministry of Public Health and Sanitation and the Director of Basic Education, Ministry of Education. Supporting letters from these ministries were sent to provincial health and education officers, detailing the purpose of the survey, survey timetable and procedures. Upon arriving in a province, meetings were held with the Provincial Medical Officers and the Provincial Directors of Education. These offices provided further letters of support to relevant district authorities and in each district, meetings were held with relevant district health and education officials.

### Surveys procedures

Selected children were asked to provide a finger-prick blood sample, which was used to assess *Plasmodium *infection in the peripheral blood and haemoglobin concentration. Children had both a RDT, which gave an on-the-spot diagnosis, and provided thick and thin blood films for microscopy. The RDT used differed according to survey phase (see Figure 1 and Table 1). The majority of children were tested with either a ParaCheck-*Pf *device or a ParaCheck-*Pf *dipstick [25], these tests are able to detect *P. falciparum*. During the September-October 2008 surveys on the coast, the RDT used was OptiMAL-IT [26] able to detect *P. falciparum *and other, non-falciparum plasmodia species. For surveys conducted in January-March 2010, the main RDT used was CareStart Malaria Pf/Pv Combo [27] which can detect both *P. falciparum *and *P. vivax*. Prior to use, RDTs were stored at room temperature and transported to the school in a cooler box and the desiccant in the RDTs was inspected for colour changes before use, and the RDT discarded if the colour had changed. Children with positive RDTs and documented fever were provided with artemether-lumefantrine (Coartem, Novartis, artemether 20 mg/lumefantrine 120 mg) according to national guidelines.

**Table 1:** The number of schools and number of school children by study phase, malaria transmission zone, age group, sex, malaria rapid diagnostic test (RDT) used, included in school malaria surveys in Kenya, 2008-2010.

	Schools	N children (%)
**Study phase**		
Sept-Oct 2008	65	6,884 (13.8)
Feb-March 2009	54	5,694 (11.4)
June 2009-March 2010	361	37,397 (74.8)
		
**Malaria transmission zone**		
High transmission lakeside	80	8,186 (16.4)
Western highland epidemic	100	10,819 (21.7)
Coast moderate risk	95	10,172 (20.4)
Central low risk	110	11,275 (22.6)
North eastern semi arid	95	9,523 (19.1)
		
**Age group**		
5-9 years		12,338 (24.7)
10-15 years		33,650 (67.3)
> 15 years		3,763 (7.5)
Missing^1^		224 (0.5)
		
**Sex**		
Male		25,656 (51.3)
Female		24,217 (48.5)
Missing^1^		102 (0.2)
		
**RDT test**		
CareStart Malaria Pf/Pv Combo	96	9,064 (18.2)
OptiMAL-IT	71	7,801 (15.6)
ParaCheck device	246	26,326 (52.8)
ParaCheck dipstick	67	6,700 (13.4)

^1 ^Data was not recorded

In all 480 schools, thick and thin blood smears were also prepared for each child. Slides were labelled and air-dried horizontally in a carrying case in the field, and stained with 3% Giemsa for 45 minutes at the nearest health facility when the teams returned from the school. Due supply difficulties in securing Hemocue curvettes for all schools, haemoglobin concentration was assessed in 399 schools and estimated to an accuracy of 1 g/L using a portable haemoglobinometer (Hemocue Ltd, Angelhölm, Sweden). Children identified as severely anaemic (haemoglobin levels < 70 g/L) were referred to the nearest health facility for treatment according to national guidelines. Transportation costs were provided and an agreement was reached with facilities to waive drug costs.

A questionnaire was administered to pupils to obtain data on mosquito net ownership and use and when treated, recent travel history, recent history of illness, key socio-economic variables such as household construction, education level of the child's guardian and ownership of household items such as mobile phones. An additional questionnaire was administered to the head teacher to collect information on ongoing school health activities, including malaria control, as well as information, education and communication (IEC) material on malaria. The pupil and school questionnaire data will be used in future analyses. The geographical location of each school was determined using a Garmin eTrex global positioning system [28].

### Expert microscopy

Blood smears of all RDT-positive children, where available, and an equivalent number of randomly selected blood slides from RDT-negative children were examined by expert microscopy either at the KEMRI-Wellcome Trust laboratory in Kilifi or the KEMRI laboratory in Nairobi. Parasite densities were determined from thick blood smears by counting the number of asexual parasites per 200 white blood cells (or per 500 if the count was less than 10 parasites/200 white cells), assuming a white blood cell count of 8,000/μl. A smear was considered negative after reviewing 100 high-powered fields. Thin blood smears were reviewed for species identification. Two independent microscopists read the slides, with a third microscopist resolving discrepant results (see Additional file 2 for microscopy results flowchart). Of the 6,655 slides examined, the overall sensitivity and specificity of the RDTs was 96.1% (95% CI: 95.2-96.9) and 61.6% (95% CI: 60.2-63.0). Diagnostic performance was similar for three types, but very poor for CareStart: 94.9% sensitivity and 77.4% specificity for OptiMal; 96.2% sensitivity and 68.7% specificity for Paracheck device; 96.3% sensitivity and 76.0% specificity for Paracheck dipstick; and 100% sensitivity and 2.0% specificity for CareStart. In light of the poor performance of CareStart, we only present slide-corrected RDT results. A more detailed investigation of the reliability of RDTs in the context of school-based malaria surveillance is the subject of future work.

### Electronic data capture

Children's responses were entered electronically in the school on either ASUS Eee PC 1005P or Acer Aspire One d250 netbook computers using a customized Microsoft Access database, which included in-built checks to prevent some errors altogether and immediately prompting for resolution of other errors. Computers were powered by batteries, backed up by solar panels or small diesel generators. At the end of each day, interview data were combined with parasitological data and transmitted nightly to Nairobi using a mobile phone modem connection. In some parts of northern Kenya, delays of 1-2 days were experienced in transmitting the data due to poor network coverage.

### Data analysis

Data were analyzed using STATA version 11.0 (STATA Corporation, College Station, TX, USA). The locations of schools were linked with survey data and mapped using Arc GIS 9.2 (ESRI, Redlands, CA, USA).

Anaemia was defined as a haemoglobin concentration <130 g/L for male children above 15 years, <120 g/L for children aged 12-14 years and female children above 15 years, <115 g/L for children aged 5-11 years and <110 g/L for children aged less than five years, with adjustment made for altitude of the school [29]. Severe anaemia was defined as a haemoglobin level <70 g/L.

Results were adjusted for clustering at the school-level using random effects regression modelling [30]. Specifically, national- and province-level estimates of *Plasmodium *infection and corresponding 95% binomial confidence intervals (CI) were estimated using a zero inflated Poisson (ZIP) model to account for the excess of schools with zero prevalence. The ZIP model was favoured over a standard Poisson model on the basis of the Vuong test [31]. The ZIP model was used for all the provincial level estimates of *Plasmodium *infection except for Nairobi and Rift Valley provinces where a standard Poisson model was used. National and Province-level estimates of anaemia and net use were estimated using generalized linear and latent mixed models (GLLAMM) adjusted for clustering at the school level.

The overall financial cost of the survey was estimated from the project accounting system, with costs divided into staff, transport, operating costs and consumables.

### Ethical considerations

The study protocol received ethical approval from the Kenya Medical Research Institute and National Ethics Review Committee (numbers 1407 and 1596). Additional approval was provided by the Permanent Secretary's office of the Ministry of Education (MoE) and the Division of Malaria Control, Ministry of Public Health and Sanitation. All national, provincial and district-level health and education authorities were briefed about the survey purpose and selected schools. Official letters of support were prepared by Provincial MoE officers.

Head teachers were briefed about the survey and were provided with an information sheet detailing the survey procedures and asking for their permission to have their school involved in the survey. The head teachers were also asked to inform the students, parents and the school committee members about the survey and obtain their approval for the study. Parents/guardians who did not want their children to participate in the study were free to refuse participation. If a parent or guardian chose not to allow their children to participate in the survey, the child's name was removed from the school rolls. On the survey day, the survey team leader informed all children in the school about the sampling and survey procedures, making it clear to their participation was voluntary and that they may opt out of the testing at any time if they choose to. After randomly sampling the students from the classrooms, individual assent was also obtained from the children before samples were collected. Very few children refused to participate in the survey and therefore replacement sampling was not required. Individual written parental consent was not sought since the survey was conducted under the auspices of the Division of Malaria Control, Ministry of Public Health and Sanitation, which has the legal mandate to conduct routine malaria surveillance, and because only routine diagnostic procedures were undertaken.

## Results

### Survey process

The surveys were carried out in two main phases (Figure 2 and Table 1): first, two independent surveys, September 2008 to March 2009; and second, a purposively selected sample of 361 schools, June 2009 to February 2010. Up to five separate teams were in the field at any one time, including up to 24 laboratory technicians. These were either recruited locally in each province from the Ministry of Public Health and Sanitation's Division of Vector Borne and Neglected Tropical Diseases, or recruited in Nairobi from KEMRI's Eastern and Southern Africa Centre of International Parasite Control (KEMRI/ESACIPAC). The majority technicians had prior experience of carrying out school surveys. Mobile telephone coverage was available throughout most of Kenya, enabling sending of data to Nairobi on a daily basis.

The average cost of surveying one school was estimated to be US$ 1,116. The largest cost component was staff (32.0%), following by transport (26.9%). Operating costs included laboratory consumables, courier services and hiring of mini-laptop computers and accounted for 24.7% of total costs. Other costs included slide reading (7.2%) and administration costs (9.2%).

### Characteristics of study participants

A total of 49,975 children in 480 schools across Kenya were included in the survey. Table 1 presents the characteristics of the study children and their schools. In each school, an average of 103 (range 23 - 115) children was selected, with an equivalent number of boys and girls sampled (51.3% boys). The median age was 11 years (inter-quartile range: 10-13 years) and most children (67.3%) were in the 10 to 15 age group. The majority (74.8%) of schools were surveyed during the second phase of the surveys, June 2009-March 2010. Data on malaria infection and ITN use were collected in all schools, whereas haemoglobin concentration was assessed in 399 schools.

### Malaria infection

**Figure 3:**
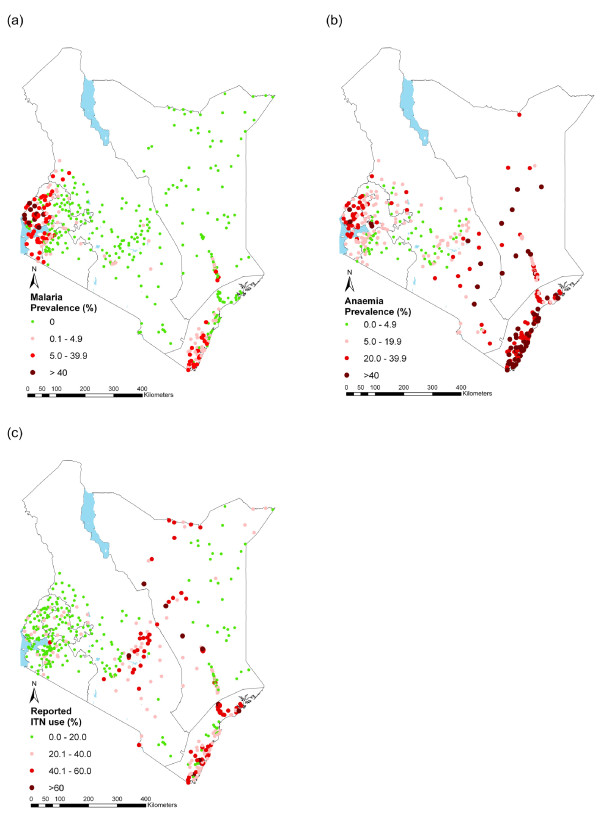
**The geographical distribution of (a) Malaria infection in 480 schools, (b) anaemia adjusted for age, sex and altitude in 399 schools, and (c) reported insecticide net use among school children in 480 schools across Kenya, September 2008-March 2010**. Note: Haemoglobin was not assessed in some schools in the North Eastern Kenya. Classification based on the WHO categories of anaemia for public health importance (WHO, 2001).

The overall prevalence of infection, based on slide-corrected RDT positivity, was 4.3 (95% CI, 3.3 - 5.2). The vast majority (96.8%) of these infections were *P. falciparum*, with the remainder being either *P. ovale *(0.1%) or *P. malariae *(0.6%) or mixed infections (2.6%); no *P. vivax *was detected. Prevalence was significantly higher in children aged 5-9 and 10-14 years old (4.4%) than children older than 15 years (2.8%, p < 0.0001), but did not significantly differ between males and females (4.3% vs. 4.2%, p = 0.53). The prevalence of malaria infection by province is shown in Table 2 and the geographical distribution of malaria is shown in Figure 3a. Prevalence varied markedly by school (0 - 70.9%) and by province, being highest in Western Province (21.6%, 95% CI: 14.6 - 28.7%) and lowest in Central and North Eastern provinces, where no child was found to be infected in any school (Table 2). Prevalence was <5% in all other provinces, except Nyanza Province (9.3%, 95% CI: 6.8 - 11.9%). Eleven (2.3%) schools had a parasite prevalence ≥ 40% and all of these were located around Lake Victoria (Figure 3a).

**Table 2:** The prevalence of malaria infection based on RDTs alone and blood slide corrected RDT results in primary school children by province in Kenya, 2008 - 2010.

	*Plasmodium *spp.						
	
	**N**^**1**^	Malaria Prevalence by RDTs (%, 95% CI)^2^	Malaria Prevalence: slide corrected (%, 95% CI)^3^	Slide corrected prevalence category (n, %)			
				
				0%	0.1-4.9%	5-39.9%	≥40%
**Total**	480/49,891	7.6 (6.4 - 8.9)	4.3 (3.3 - 5.2)	296 (61.7)	98 (20.4)	75 (15.6)	11 (2.3)
**Province**							
Nyanza	90/9,299	19.6 (16.0 - 23.1)	9.3 (6.8 - 11.9)	30 (33.3)	25 (27.8)	31 (34.4)	4 (4.4)
Western	37/3,892	32.4 (25.2 - 39.6)	21.6 (14.6 - 28.7)	5 (13.5)	8 (21.6)	17 (45.9)	7 (18.9)
Central	22/2,387	0	0	22 (100)	0	0	0
Rift Valley	87/9,202	1.2 (0.7 - 2.0)	0.8 (0.4 - 1.5)	66 (75.9)	16 (18.4)	5 (5.7)	0
Nairobi	10/917	1.9 (1.2 - 3.0)	1.1 (0.6 - 1.6)	3 (30.0)	7 (70.0)	0	0
Eastern	52/5,355	0.2 (0.1 - 0.4)	0.1 (0.0 - 0.2)	49 (94.2)	3 (5.8)	0	0
North Eastern	43/4,087	1.0 (0.5 - 1.5)	0	43 (100)	0	0	0
Coast	139/14,752	3.8 (2.9 - 4.6)	2.3 (1.7 - 2.9)	78 (56.1)	39 (28.1)	22 (15.8)	0

^1 ^Number of schools surveyed/children tested, excluding schools without microscopy results.

^2 ^Prevalence and 95% binomial confidence (CI) intervals were calculated using a zero inflated Poisson model adjusted for clustering at the school level, except in Nairobi, Rift Valley and Eastern provinces where a random effects cluster adjusted Poisson model was used.

^3 ^Prevalence and 95% binomial confidence (CI) intervals were calculated using a zero inflated Poisson model adjusted for clustering at the school level, except in Nairobi and Rift Valley provinces where a random effects Poisson model was used.

### Anaemia

The overall prevalence of anaemia was 14.1% (95% CI: 13.0-15.3%) and the mean haemoglobin concentration was 128.8 g/L (95% CI: 127.9-129.7 g/L). Anaemia was more common among children aged 15 years and above (38.6%, 95% CI: 33.1-44.9%) than 10-14 years (14.9%, 95% CI: 12.6-17.5%) and 5-9 year olds (14.0%, 95% CI: 14.1-18.1%). There was no difference in prevalence of anaemia among males and females (13.3% (95% CI: 12.1-14.7) vs 13.3 (95%CI: 12.0-14.8)). Anaemia varied markedly by school (0-75%, figure 3b) and was more common in Coast Province and least common in Central Province (Table 3).

**Table 3:** The prevalence of anaemia and the proportion of school children reporting using and sleeping under a long-lasting insecticide net the previous night by province in Kenya, 2008-2010.

	**Anaemia**		**Any net**			**Insecticide-treated net**		
			
	**N**^**1**^	**Prevalence** **(%, 95% CI)**^**2**^	**N**^**1**^	**Reported use** **(%, 95% CI)**^**2**^	**Sleeping under previous night** **(%, 95% CI)**^**2**^	**N school with <20% use** **(n, %)**	**N school with ≥60% use** **(n, %)**	**Overall use** **(%, 95% CI)**^**2**^
		
**Total**	399/41920	14.1 (13.0-15.3)	480/49,797	44.2 (42.7-45.6)	42.1 (40.9-42.8)	264 (55.0)	11 (2.3)	19.0 (18.0-20.2)
**Province**								
Nyanza	85/8969	11.3 (9.6-13.3)	90/9,316	39.9 (38.2-41.5)	38.5 (36.8-40.1)	73 (81.1)	0	13.5 (12.1-15.0)
Western	35/3751	19.8 (15.7-25.1)	37/3,892	44.1 (42.1-46.0)	41.1 (38.0-44.2)	30 (81.1)	0	13.3 (11.2-15.6)
Central	22/2387	3.6 (2.3-5.4)	22/2,387	37.8 (36.2-39.5)	37.1 (35.4-38.7)	14 (63.6)	2 (9.1)	10.8 (8.6-13.5)
Rift Valley	63/6629	6.8 (5.5-8.3)	90/9,202	29.7 (27.7-31.7)	28.7 (27.0-30.4)	71 (78.9)	0	9.5 (8.2-11.1)
Nairobi	10/924	5.2 (3.7-7.4)	10/919	44.7 (41.4-48.1)	38.6 (34.8-42.5)	8 (80.0)	0	17.3 (12.3-24.6)
Eastern	33/3399	12.4 (9.8-15.5)	52/5,355	69.4 (67.1-71.7)	55.2 (52.8-57.5)	5 (9.6)	2 (3.8)	40.0 (36.9-43.4)
North Eastern	13/1262	32.8 (26.7-40.4)	43/4,078	60.1 (57.9-62.2)	50.5 (48.0-53.0)	32 (74.4)	2 (4.7)	4.1 (3.1-5.4)
Coast	138/14599	44.6 (39.8-49.9)	139/14,648	66.7 (65.1-68.4)	62.6 (60.5-64.7)	31 (22.3)	5 (3.6)	35.5 (33.0-38.1)

^1 ^Number of schools surveyed/children tested.

^2^Prevalence and 95% binomial confidence intervals calculated using generalized linear and latent mixed models (GLLAMM) adjusted for clustering at the school level.

### Reported ITN use

Overall, 44.2% (95% CI: 42.7-45.6%) of children reported having a bed net and 42.1% (95% CI: 40.9-42.8%) reported sleeping under a net the previous night. However, of the children asked about sleeping under an ITN, less than a quarter (19.0%, 95% CI: 18.0-20.2%) reported sleeping under an ITN while 6.4% did not know whether their nets were ITNs or not. The majority (70.9%, 95% CI: 68.6-73.2%) of nets, non-ITNs or ITNs, were reportedly obtained from the health facilities. Reported use of ITNs varied markedly across the country (Figure 3c), and was <20% in the majority (55.0%) of schools, especially in Nyanza and Western Provinces; disappointedly, only eleven schools had reported ITN use >60% (Table 3). In terms of household net ownership, 80.3% of children reported having at least one bed net in their household while 77.1% reported more than one net in their households. Nyanza Province had the highest number of children reporting having at least one net in their household (88.9%) while Central Province had the lowest (57.1%).

### Fever and absenteeism

Overall, 13.5% of children reported a fever on the day of the survey, but of the children that had their axillary temperature measured only 733 (2.4%) children had a temperature >37.5°C. Of these febrile children, only 55 (7.5%) children had a malaria infection. Of the children asked about their absenteeism history (n = 37,288), 26.9% of children reported being absent from school due to illness for at least one day in the last two weeks, with the commonest cause of illness being headache (56.7%), whilst 17.0% reported malaria as the cause for absenteeism.

### Malaria control activities

A comprehensive school level questionnaire was administered in 344 schools, predominantly in Western, Central, Rift Valley and Nyanza provinces during the second phase of the survey. Of these schools, only 59 (17.2%) reported having had any malaria control activities, such as indoor residual spraying of the school buildings and draining of stagnant water, in the last 12 months and the majority (21) of these schools were located in the malaria high transmission zone. Only, seven schools had malaria IEC materials in at least 1 classroom, 8 had IEC materials in the head teacher's office while 17 schools had IEC booklets in the school library.

## Discussion

Reliable, contemporary data are essential prerequisites for the planning and implementation of effective malaria control. Each national programme needs to be tailored to its specific national context, based on a cartographic understanding of malaria transmission intensity and current intervention coverage. The data from the present study show that although the national prevalence of *Plasmodium *infection was relatively low at 4.3%, there existed marked variation across the country. This finding is consistent with national level malaria prevalence estimate observed in the Kenya MIS in 2007, where the malaria prevalence by RDTs was 7.6% and 3.4% by microscopy [32].

The observed distribution of low transmission in most of Nairobi and Central provinces and some parts of the Eastern and Rift Valley provinces and high transmission along the shores of Lake Victoria and the south coast is consistent with a recent model of malaria risk in Kenya [33]. The current findings also highlight marked variation in the levels of reported ITN use, with levels highest in Eastern and Coast provinces, but surprisingly low reported levels in western Kenya. Interestingly, this low coverage in western Kenya contrasts findings from recent nationwide household cluster surveys among all ages carried out between 2007 and 2009, which showed that ITN/LLIN coverage was similarly high in the western, coastal and central regions of the country [34]. This suggests that in western Kenya, although overall coverage in the community is high, school children are not using ITNs. Possible reasons for the low ITN use among school children have been discussed elsewhere [35,36], but are likely to reflect a previous focus of net distribution programmes on providing nets to young children and pregnant women. An additional explanation may lie in household sleeping patterns with school children sleeping separately from their younger siblings and parents in areas, such as kitchens, where nets cannot readily be hung [37].

The current survey took advantage of the existing school infrastructure and historical experience in carrying out school surveys in Kenya to achieve a rapid, inexpensive approach to malaria surveillance. The adopted approach has a number of advantages over malaria indicator surveys (MIS) which provide nationally-representative household survey data on coverage of malaria interventions as well as malaria parasitaemia and anaemia among household members most at risk, namely children under five years and pregnant women [9]. First, school surveys make use of an annually updated national school database as a sampling frame, rather than a list of Enumeration Areas (EAs) or clusters from population censes which are typically conducted every 10 years. Second, sampling of children in schools is greatly simplified as children are easily identified from the school register. Third, estimates of parasite rate among school-aged children can be more readily age-standardised to the optimal 2-10 years estimate of *Pf*PR than estimates among under fives and pregnant women [12]. Finally, the costs are greatly reduced: the average cost of surveying one school was estimated to be US$ 1,116. These costs compares to an estimated cost of US$ 3,299 cluster sampled in the Kenya 2007 MIS (Division of Malaria Control, Nairobi, Kenya. Personal communication); however, these estimates represent only financial costs and a detailed economic cost analysis of alternative survey approaches is clearly warranted.

School malaria surveys are not without their limitations, however (for a review see [12]), and a number are highlighted here. First, the representativeness of school surveys will depend on the level of school enrolment. In Kenya, net enrolment rates are lowest in North Eastern Province (27.5%), Nairobi (44.9%) and Coast Province (71.8%) and, therefore, school surveys may not provide a truly representative picture of malaria among school-aged children in these provinces. In the remaining provinces, however, net enrolment rates exceed 90%, increasing the representativeness of school surveys. A further way in which school surveys may be unrepresentative is that children found to be absent on the day of the survey, and therefore not included in the sample population, may be absent due to illness, including malaria. In the current surveys, 26.9% of children reported being absent for at least a day in the two weeks preceding the survey. Due to logistical constraints, no effort was made to follow-up absent children, thus introducing potential selection bias. This may be a particular problem in areas of low malaria transmission, where infection generally leads to clinical disease; whereas in high transmission areas, the majority of infections will be asymptomatic with many of infected children present in school. This issue of potential sampling bias and how it varies according to malaria endemicity deserves further investigation. However, if school surveys underestimate true prevalence of infection in the wider community by a consistent amount, which can be calibrated, schools may still provide a promising platform for malaria surveillance.

A further drawback of school surveys is that they cannot provide complete information on household ownership of insecticide-treated mosquito nets and their use by children under five years of age and pregnant women, or on the use of the intermittent preventive treatment during pregnancy and the type and timing of treatment of fever in children under five years of age. However, a study in Uganda found that reports by schoolchildren on household net ownership provide a rapid method to collect reliable coverage data at the community level [38].

There are also several practical features of the present survey worth highlighting. First, the survey used modern technology to achieve a more cost efficient approach to data collection. In particular, data captured were achieved using netbook computers with customized data entry screens. Electronic data capture systems, mainly based on the use of personal digital assistants (PDAs), are shown to be acceptable and reduce data entry errors considerably [39-42]. Experience in the use of laptops or netbook computers is more limited, but a recent study comparing PDAs and laptops for data capture found the use of laptops was associated with fewer typing errors and missing data [43]. Further use of laptops or netbook computers for data capture in settings where tables can be found, such as schools and health centres, is strongly encouraged.

Second, consent for the survey was based on a passive, opt-out method of parental permission. This approach is considered to be an ethical and practical way of informing participants in low-risk studies and interventions [44], and has been used in a number of school-based studies, including studies in the United Kingdom, United States and India [45-48]. Such consent procedures, when compared to the opt-in methods of seeking parental consent, reduce the time needed to seek consent and maximize participation therefore avoiding significant sampling bias and under-reporting.

Third, malaria parasitaemia was ascertained using a two-stage approach of malaria RDTs and blood slides. Importantly, this approach enabled appropriate treatment of clinical malaria in schools on the day of the survey, but also allowed assessment of the reliability of RDTs at a later stage. A drawback of using RDTs is that they can result in false positives, especially those RDTs that detect the histidine-rich protein-2 (HRP-2) antigen [49-51], leading to an overestimation of infection prevalence [52]. Such overestimation of prevalence using RDTs may lead to misclassification of schools in low and moderately high prevalence, however the effect of such systematic misclassification on resource allocation for malaria control remains unclear and will be the subject of further work. Encouragingly however the observed sensitivity of RDT in the present study exceeded 90% and are consistent with findings of a recent WHO-FIND evaluation [50].

In conclusion, the current study describes recent experiences of school malaria surveys in Kenya and highlights the potential of school surveys as a complementary approach for malaria surveillance to MIS. The adopted survey approaches evolved over time and important practical lessons were learnt which can inform the conduct of future school malaria surveys. Notwithstanding, there remain several issues requiring further investigation, including the representativeness of schools, the reliability of school children's reports of ITN ownership and use, the reliability of RDTs among school children. It is hope that addressing these issues will provide clearer direction on the role, in Kenya and elsewhere, of schools in an integrated national malaria surveillance system, which also includes household surveys and health facility reporting.

## Competing interests

The authors declare that they have no competing interests.

## Authors' contributions

CWG participated in the data collection, analysis and developed the draft manuscript. PNK, JK and MM were responsible for fieldwork supervision and contributed to the final manuscript. EJ, RWS and AN were responsible for the study design, interpretation and scientific guidance. SB was responsible for the overall project management, study design, scientific guidance and writing of the manuscript. All authors read and approved the final manuscript.

## Supplementary Material

Additional file 1 **Field manual for school malaria surveys, Kenya**. The field manual developed for the national school malaria survey and used the field teams. Click here for file

Additional file 2 **Microscopy results flowchart**. A flow chart showing the numbers of slides examined and the microscopy results, including discrepant results, in the school malaria surveys in Kenya, 2008-2010. Click here for file
