# Supplementary material for: Implementing school malaria surveys in Kenya: towards a national surveillance system
Source: Malar J. 2010 Oct 30;9:306. doi: 10.1186/1475-2875-9-306 (PMC2984573; doi:10.1186/1475-2875-9-306)
Supplement: Additional file 1 — Field manual for school malaria surveys, Kenya. The field manual developed for the national school malaria survey and used the field teams. [file 1475-2875-9-306-S1.PDF]

KENYA SCHOOL MALARIA SURVEY

FIELD MANUAL

2009-2010

Kenya Medical Research Institute-Wellcome Trust Research Programme
P.O. Box 43640 – 00100
Nairobi, Kenya

Email: simon.brooker@lshtm.ac.uk

Contents

1. Introduction 4
Aim of the study 4
Study outline and main outcomes 4
Purpose of the manual 5
2. Survey procedure 6
Survey team 6
Survey preparation 6
Consenting 6
Selecting children 7
Survey procedures 7
3. How to complete the questionnaire 9
Overview of the questionnaire 9
Completing the questionnaire 9
Section 1: Child information 9
Section 2: Health information 10
Section 3: Residence 11
Section 4: Family and siblings 11
Section 5: Household wealth information 11
Section 6: Bednet use and IRS 11
Section 7: Deworming use 11
Section 8: Absenteeism and recent illness 11
Section 9: Travel history 12
Appendix 1: Daily supplies list 13
Appendix 2: Random number table and child selection 15
Appendix 3: Child Information form 16
Appendix 4: Child Questionnaire 17
Appendix 5: Teacher questionnaire 21
Appendix 6: School questionnaire 24
Appendix 7: Survey information sheet 26
Appendix 8: Malaria blood slide preparation 28
Appendix 9: Staining of blood slides 30

Appendix 10: Collecting filter paper blood spots for the recovery of human antibodies 31
Appendix 11: Haemoglobin assessment using HemoCue device 37
Appendix 12: Preparation of the malaria rapid diagnostic test (RDT) 38
Appendix 13: Kato-Katz preparation 40
Appendix 14: Urine filtration preparation 42
Appendix 15: Weight dosage charts 44
Artemether-lumefantrine 44
Albendazole: 44
Praziquantel: 44
Appendix 16: Data entry on Ms Access database 45
1. Guidelines for laptop care and maintenance 45
2. Using the KNSS database 45
3. Merging data from several computers 49
4. Exporting merged data 50
Appendix 17: Definitions and Abbreviations 54
Definitions 54
Abbreviations 54

1. Introduction

Malaria remains a major public health problem in Kenya, however the epidemiology of malaria risk is changing following considerable investment in malaria control activities since 2001. In January 2009, the Division of Malaria Control (DOMC) of the Ministry of Public Health and Sanitation began the process of reviewing the national malaria control programme with the aim of developing a new National Malaria Strategy for the next ten years. As part of this review, the DOMC has recognized the need to tailor its malaria control interventions to the individual needs of districts based on malaria risk and target specific population sub-groups to achieve an effective and sustainable universal coverage. Several strategies, including new initiatives such as home-based management of malaria and control of malaria in schools under a ***Malaria-free schools initiative*** have been proposed.

In 2005, the Kenya education sector constituency initiated the ***Kenya Education Sector Support Programme (KESSP)***, whose overarching goal is of enhancing access, equity, and quality at all levels of education and training. To realise this, the programme has 23 investment programmes, one of which is the ***School Health, Nutrition and Feeding Investment Programme***. The aim of this programme is to provide school-based health, with an initial focus on school-based treatment for soil-transmitted helminths (STH) and schistosomiasis. Future plans include the implementation of malaria control through schools.

To guide these government efforts, the Kenya Medical Research Institute-Wellcome Trust Research Programme has been requested by the DOMC and the World Health Organization's Kenya office to implement a national school malaria survey in Kenya.

Aim of the study

To assess malaria parasite infection and anaemia in Kenyan school children, and their use of malaria control interventions and travel histories.

Study outline and main outcomes

A representative national sample of 533 public primary day, mixed schools has been selected from a national sampling frame of over 19,177 schools. This sample has been selected to represent the spatial distribution of schools with 3-5 schools selected per district. After consenting, each sampled child in these schools will be:

- Tested for malaria using malaria rapid diagnostic test (RDT)
- Tested for Haemoglobin using a Hemocue machine
- Asked questions on bed net use and the overall bed net use in their household.
- Asked questions on recent travel history
- Asked questions on recent de-worming
- In selected schools, children will also be tested for Soil-transmitted helminths (STH) as well as urinary and intestinal schistosomiasis.

Basic child information will be recorded on a child information form, to keep track of the children during the survey, and interview data will be entered directly in the school on to mini-computers using customised Microsoft Access data entry screens. All the steps and details of procedures are detailed in the standard operating procedures (SOPs) included in this manual. It is essential to adhere to universal precautions for handling blood and potentially infectious materials for the safety of both the participating children and members of the survey team.

Purpose of the manual

This manual is intended to guide the survey team leaders, technicians and the field interviewers on the general organisation of the survey and specific survey procedures. The quality of your work will impact on the overall quality and usefulness of the results. Therefore, it is essential that you clearly understand what you are expected to do. After you have studied the manual, you should be able to:

1. Understand the purpose and outline of the study;
2. Have a clear idea of the procedures of patient enrolment and documentation;
3. Prepare and stain thick and thin blood smears;
4. Prepare blood spots on filter paper;
5. Prepare, read and interpret malaria rapid diagnostic tests (RDTs);
6. Prepare kato-katz thick faecal smears and read for STH infection and intestinal schistosomiasis;
7. Undertake urine filtration for the detection of urinary schistosomiasis;
8. Store study slides and filter papers appropriately;
9. Dispose of infectious waste materials appropriately;
10. Know where the find the appropriate information in the guide when needed.

2. Survey procedure

Survey team

The survey team will include the following:

Team members	Tasks
Team leader	Overall team coordination and organisation
Education official	Introducing the team to the school and child registration
Laboratory technicians	Sample collection, preparation and reading of the results.
Interviewers	Data collection and entry.

In addition to the tasks outlined above, the teams members are expected to work as part of a team and perform any other tasks as may be requested by the team leader.

Survey preparation

Prior to the survey, the team leader should do the following:

- Inform the Provincial Director of Education (PDE) and the Provincial Director of Public health and Sanitation (PDPHS) about the survey – if possible, the survey team leader should request the PDE to write a letter to the District Education Officers (DEOs) informing them of the survey;
- Inform the DEO and District Medical Officer of Health (DMOH) about the survey and request the DEO for an education official to accompany the team to the schools;
- Through the education official inform all the sampled schools about the survey and the survey dates.
- Issue the schools with the survey information sheet (Appendix 7);
- Ask the head teacher to inform the students and parents about the survey;
- Ask the head teacher to provide a room, chairs and tables (if available) to be used during the survey.
- The survey team leader should also ensure that the daily supplies are available before setting off to the school each day. Use the daily supplies list in **Appendix 1**.

Consenting

The survey team leader should contact the school before the survey and ask the head teacher to inform the parents, the PTA and the students about the survey. On the survey day, the team leader should explain the survey objectives and seek consent to conduct the survey from the school head teacher.

Parents/guardians who do not want their children to participate in the study are free to refuse participation. If a parent or guardian chooses not to allow their children to participate in the survey, the child's name should be removed from the school rolls before random selection. On the survey day, the survey team leader should inform all children, in Swahili or the local language, in the school about the sampling and survey procedures, making it clear to their participation is voluntary and that they may opt out of the testing at any time if they choose to.

Selecting children

Each team will be provided with tables of random numbers (**Appendix 2**) which are to be used to randomly select children. Using the table of random numbers, the survey team leader should randomly sample 22 students from each class (11 boys and 11 girls) from classes 2 - 6, each class at a time to minimize disruption.

In selected schools, sampled children are to be issued with pre-labelled stool and urine containers to collect the urine and the stool samples. The containers should be labelled with the Child ID using a permanent marker.

Survey procedures

The classroom should be organised in such a way that the students start with the registration, then proceed to sample collection, and finish with the interviews. (See diagram below)

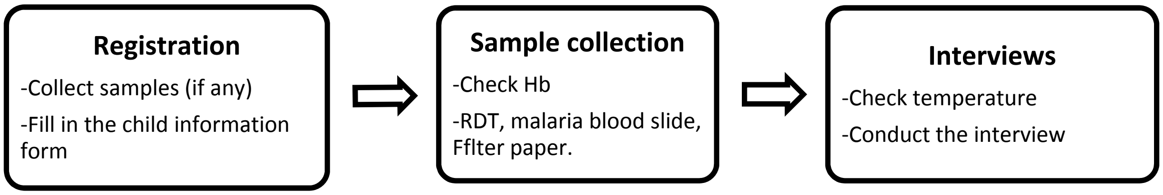

a) Registration

1. Where appropriate, collect stool sample and urine sample. Ensure that the samples are correctly and clearly labelled with the child ID (see Section 3).
2. Complete the child information form (**Appendix 3**) making sure that the child ID on the form matches the child ID on the collected samples.

b) Sample collection

3. Prepare and label blood slides, filter paper and the RDTs (**Appendix 8 – 12**)
4. Take finger prick blood sample (**Appendix 8**).
5. Use blood sample for haemoglobin reading (**Appendix 11**).
6. Prepare thick and thin blood smears on a single slide (**Appendix 8**).
7. Collect blood spot on filter paper (**Appendix 10**).

c) Interviews

8. Temperature measurement and recording.
9. Enter the information on the child information form (**Appendix 3**).
10. Complete remaining sections of questionnaire on the min-laptops (**Appendix 16**). Data should be checked for completeness by team leader before leaving the school.
11. Complete the teacher questionnaire (**Appendix 5**) by interviewing the head teacher and the health teacher- to be done by the team leader.
12. Complete school questionnaire (**Appendix 6**) by interviewing head teacher or another member of staff – to be done by the team leader.
13. Using the geographical positioning system (GPS), record geograpical coordinates of the school on the school questionnaire.

d) Kato-Katz, urine filtration and blood slide fixation

14. Make Kato-Katz thick smear from collected stool samples (**Appendix 13**), making sure that the child ID on the sample matches the child ID on the slides.
15. Read stool smear within 1 hour for hookworm and record results (**Appendix 13**)
16. Re-read the slides for other heminth species at later time.
17. Filtrate the urine and make the slides (**Appendix 14**)
18. Read the urine slides.
19. Fix and stain blood smears.

3. How to complete the questionnaire

It is essential to ensure that the information typed into the mini-computers is consistent with the information on the child information sheet. The interviewer should ensure that the child's privacy is respected while answering questions. This is not only beneficial to the student but also the students are more likely to give honest answers if their privacy is respected.

Overview of the questionnaire

The questionnaire contains several sections, with each section aimed at collecting certain information related to child health.

Section 1: Child information. This section captures the details of the student including the school code, child ID, student name and age, and their parent's name.

Section 2: Health information. This section is to be completed after the blood testing has been completed. Auxilliary temperature is also recorded in this section.

Section 3: Residence. This section asks about the child's place of residence.

Section 4: Family and Siblings. This section captures information about the child's family and siblings.

Section 5: Household wealth information. This section records information on the education of the household head, household water sources, household construction materials, and household possessions.

Section 6: Bed net use and indoor residual spraying (IRS). This section asks questions on the child's net use. This includes details of the net (if one is present), where it was obtained, how it was treated, and the total number of nets the household possess. It also asks about recent IRS

Section 7: Deworming use. This focuses on recent deworming in the last year.

Section 8: Absenteeism and recent illness. This section asks about absenteeism during the current school term and the reasons for any absenteeism. It also asks about fever history in the last two weeks before the survey and on the day of the survey.

Section 9: Recent travel history. This section focuses on the child's travel history within the past two months and asks questions on the journey details (if any).

Completing the questionnaire

Section 1: Child information

- *Primary school ID:* All the schools will be given a unique five digit ID, which is a component of the Province, District and the school ID.

The Province IDs are as follows:

- Central - 1
- Coast - 2
- Eastern - 3

- Nairobi - 4
- North Eastern - 5
- Nyanza - 6
- Rift Valley - 7
- Western - 8

An example of a school ID;

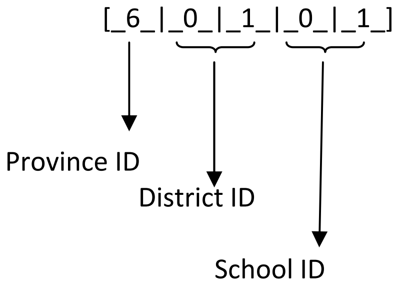

Therefore, school ID 60101 would be the first school to be surveyed in the first district in Nyanza province. **Note: All the schools are pre- coded and the pre-assigned codes should be used.**

- *Primary school name:* That is the name of the school e.g. Bogitaa.
- *District ID:* A three digit ID, which is a component of the Province ID and the District ID.
- *District name:* The name of the district.
- *Child ID:* The child ID is a component of the school ID and the child number. The students are assigned numbers from 001 to 110. Therefore, child number one in school 60101 would have the child ID 60101001.
- *Student's first and last name:* Student's names as they appear on the school register.
- *Student's initials:* These are the first letters of the student's first and last name.
- *Date of birth:* The date when the student was born. The date should be in a six digit format as follows; dd/mm/yy. If the child does not know the date of birth, enter 99/99/99.
- *Age:* The age of the student in years.
- *Gender:* Whether the student is male or female.
- *Parent / guardian's first and last name:* These are the names of the student's parent or guardian.

Section 2: Health information

This section should be filled by the laboratory technologists after taking the malaria blood slide, filter paper, malaria RDT and checking for Hb. The person at the registration point should indicate whether or not the stool sample was provided.

Section 3: Residence
A1: Enter the village name. (Make sure that the child understands the meaning of a village)

A2: Enter the walking time in minutes.

Section 4: Family and siblings
B1: Ask the student to tell you the total number of people that normally reside in their household.
B2: This question asks about the students siblings (brothers and sisters from the same mother). Enter the total number.
B3: This is a follow-up on question B2, and is asking about how many of the brothers and sisters in B2 are in school. **Do not include the respondent.**

Section 5: Household wealth information
C1: Enter the highest level of education attained by the household head. Choose on the drop down menu by clicking on the relevant option.
C2: Enter the type wall in the household head's house. Choose from the drop down menu. If the wall type is not among the choices provided, enter `others' and specify what others is.
C3: Choose from the drop down the type of flooring in the household heads house. If other, specify.
C4: Enter the **main** source of drinking water. Some household's may have more than one source of water, in this question; enter only the main source of water.
C5: Ask the child if there are any of the listed things in their household and enter yes or no accordingly.

Section 6: Bednet use and IRS
D1: This question asks if the child usually sleeps under a bed net.
D2: You should ask this question even if the child does not have a net in D1.
D3 – D8: These questions are asking about the details of the usual bed net in D1. They ask about the colour of the net, when it was obtained, the source and also about net treatment. In question D6, if a net is pre-treated then the answer to the question should be `yes'.
D9: Asks about the total number of nets available in their household whether in use or not. But these should be nets in a usable condition.
D10: This question asks if the household has been sprayed with an Indoor residual spray. Make sure that the child understands that the question is asking about an IRS but not the aerosol sprays.

Section 7: Deworming use
E1: This question asks if the child has received any treatment for worms in the last one year.
E2 – E4: These questions ask about the treatment details i.e. the treatment source, number of tablets given and the colour.
Note: If the students say that they were de-wormed in school, confirm with the health teacher or the head teacher if this was done in the school.

Section 8: Absenteeism and recent illness
F1: This question is concerned about absenteeism due to illness. Enter yes or no according to the response given.
F2: The question asks about absenteeism in the last two weeks prior to the survey.
F3: If the child had been sick in the last two weeks, ask them what they were suffering from and choose from the list of illness provided. The child could have had more than one illness.
F4: This question asks about fever during the day of the interview. Make sure the child differentiates between a fever and just feeling warmer than usual.
F5: Ask if the child has had a fever in the last two weeks from the day of the survey.

Section 9: Travel history

G1: Ask if the child has made any **overnight** trips during the last two months.
G2: Indicate the number of trips made within the two months.
G3: Enter the journey details.

Appendix 1: Daily supplies list

Stationery	Amount needed
Thermometers	3
Rulers	1
Pencils	3
Sharpeners	1
Erasers	1
Pens	3
A4 size notebook	2
Marker pens (fine tip)	3
**Blood**	
Hemocue machines	2
RDTs	110
Hemocue micro-cuvettes	110
Tripple timer	1
Slides	110
Blood lancets	110
Slide boxes	1
Cottonwool	1
Sharps containers	1
Methylated spirit	1
Gloves	1 box
Airtight containers	1
Plastic sealable bags (8' * 10')	1
Plastic sealable bags (small)	110
Cooler boxes (20 litres)	1
Gauze roll	1
AA size Batteries	4
Filter papers	110
Slide folders	2
**General**	
Garbage bags	2
Dustbins	2
Hand washing soap	1
Paper towels	1

**Data**	
Laptops + adapter cables + mouse	3
Power source (Generator/solar panel)	1
Extension cables	2
USB flash	1
Internet dongle	1
Spray duster (Gas duster)	1
GPS	1
Child information forms	110
School questionnaire	1
Teacher questionnaire	2
**Other**	
Anti-malarials	Sufficient quantities
Anti-pyretics	

Appendix 2: Random number table and child selection

Class 2_Boys	Class 2_Girls	Class 3_Boys	Class 3_Girls	Class 4_Boys	Class 4_Girls	Class 5_Boys	Class 5_Girls	Class 6_Boys	Class 6_Girls
5	3	2	11	5	6	7	4	9	11
9	1	3	9	11	1	2	5	11	7
8	9	2	4	8	4	2	6	5	11
6	7	2	5	5	8	3	10	3	1
2	8	5	11	4	6	11	8	3	3
5	9	6	4	11	1	3	2	9	7
2	11	7	6	2	2	11	9	9	9
6	4	5	6	1	7	10	7	11	9
1	7	3	10	6	7	11	11	1	5
7	4	7	1	8	1	10	1	7	3
2	7	11	4	11	3	11	3	1	10

The steps to select a random sample are as follows:

- Select the first class, class 2.
- Call all children to line up in no particular order into two lines, the boys in one and the girls in another.
- Use the random number table to select 10 boys plus 1 reserve. For example, if the first number is 6, then select the sixth boy in the line. If the second number is 9, then start counting from 1 until you select the ninth boy. And so on.
- Once you reach the end of the line, return to the start and repeat until you have selected 11 boys. Send these children to the registration table where they are assigned a unique ID number for the purposes of identification on all data collection forms and tests.
- Repeat this procedure for the girls.
- Once the first class is selected, repeat for the second class, and so on until you have selected children from all five classes, 110 children in total.

Each school you visit will have a unique random table number provided to the team leader.

Appendix 3: Child Information form

CHILD INFORMATION	
**Primary school code:** |_____|_____|_____|_____|_____|	**Primary school name:**
**District Code: [____]____]____]**	**District name:**
**Child** |_____|_____|_____|_____|_____|_____|_____|_____|	**Date of visit:** |____|____|/|____|____|/|____|____| ***day*** ***month*** ***year***
**Student's last name**	**Student's first name**
**Age:** |_____|_____| **years**	**Gender:** ☐ **Male** ☐ **Female**
**HEALTH INFORMATION**	
**Haemoglobin:** |_____|_____|_____| g/L	**Auxiliary temperature:** |_____|_____| . |_____| °C
**Malaria RDT used:**	**Malaria RDT:** Positive ☐ Negative ☐ Invalid ☐
**Blood slide taken:** ☐ Yes ☐ No	**Stool slide taken:** ☐ Yes ☐ No
**Filter paper #1:** ☐ Yes ☐ No	

Appendix 4: Child Questionnaire

KENYA NATIONAL SCHOOL MALARIA SURVEY, 2009

CHILD INFORMATION	
**Primary school code:** |_____|_____|_____|_____|_____|	**Primary school name:**
**District Code: [____]____]____]**	**District name:**
**Child ID |**_____|_____|_____|_____|_____|____|____|____|	**Date of visit:** |____|____|/|____|____|/|____|____| ***day month year***
**Student's last name**	**Student's first name**
**Student's initials**	**Date of birth** |____|____|/|____|____|/|____|____| ***day month year*** 99/99/99=Not known
**Age:** |_____|_____| **years**	**Gender:** ☐ **Male** ☐ **Female**
**Parent/guardian's last name**	**Parent/guardian's first name**

HEALTH INFORMATION	
**Haemoglobin:** |_____|_____|_____| g/L	**Auxiliary temperature:** |_____|_____|_____| . |_____| °C
**Malaria RDT used:**	**Malaria RDT:** Positive ☐ Negative ☐ Unreadable ☐
**Blood slide taken:** ☐ Yes ☐ No	**Stool slide taken:** ☐ Yes ☐ No
**Filter paper #1:** ☐ Yes ☐ No	

RESIDENCE
A1. What is the name of the village you reside in now? ________________________________
A2. In minutes, how long does it take you to walk to schools?.. [___ [___ [___]<br>**Enter 999, if Don't know**

FAMILY AND SIBLINGS
B1.How many people live in your household?<br>.. [___ [___]
B2. How many brothers or sisters do you have? /<br>.. [___ [___]
B 3.How many of these siblings (in B2) attend school? /<br>.. [___ [___]

HOUSEHOLD WEALTH INFORMATION
C1. What is the highest level of education attained by the household head?<br>**Read out options, only enter one answer** .... [___]<br><br>1 = No education; 2 = Primary incomplete; 3 = Primary complete; 4 = Secondary incomplete; 5 = Secondary complete or above;

Don't know

C2. What type of wall does your house have?
Read out options, only enter one answer........ [__]

1=Stone or bricks or cement; 2=Clay or mud; 3=Wood; 4=Iron sheets; 5=Other *specify* [______________________]

C3. What type of flooring is there in the household head's house?
Read out options, only enter one answer .... [__]

1=Cement or tiles or linoleum; 2=Wooden planks; 3=Earth or sand; 4=Iron sheets;
5=Other .... *specify* [______________________]

C4. What type of roof does the household head's house have?
Read out options, only enter one answer .... [__]

1=Tiles; 2=Iron sheets; 3=Grass or thatch; 4=Makuti; 5=Other (*specify*) [______________________]

C5. What is the <u>main</u> source of water for drinking or cooking in this household?
Read out options, only enter one answer .... [__]

1=Piped/tap water; 2=Borehole or well; 3=Rain water; 4=Stream or river; 5=Bought; 6=Bottled water
7=Others........ *specify* [______________________]

C6. In your house, are there any of the following?
Read out and fill with 1= Yes; 0 = N:

Electricity .... [__]
Pit latrine .... [__]
Mobile phone .... [__]

BEDNET USE and IRS

D1. Do you normally sleep under a bednet?
Read out options, only enter one answer .... [__]

1 = Yes; 2 = No

D2. Did you sleep under a bed net last night?
Read out options, only enter one answer .... [__]

1 = Yes; 2 = No

D3. What is the colour of your bed net?
Read out options, only enter one answer .... [__]

1 = Blue; 2 = Green; 3 = White; 4 = Red; 5 = Others .... *specify* [___________________]

D4. When did you receive your bed net?
Read out options, only enter one answer .... [__]

1 = This school term; 2 = Last school term; 3 = Last school year; 4=Before last school year

D5. From where did you receive your bednet?
Read out options, only enter one answer .... [__]

1 = School; 2 = Health centre; 3 = Gift; 4 = Community programme; 5 = Shop; 6= Others….. *specify* [______________________]

D6. Has the net ever been treated with an insecticide?
Read out options, only enter one answer .... [__]

1 = Yes /; 2 = No; 3 = Don't know

D7. If yes in D6, where was the net treated?
Read out options, only enter one answer .... [__]

1 = Pretreated net; 2 = Treated at home; 3 = Don't know; 4 = Other Specify [_________________________________]

D8. If yes in D6, when was the net last treated?
Read out options, only enter one answer .... [__]

1= Pretreated net; 2 = Within the last 6 months; 3 = Not within last 6 months; 4 = Don't know

D9. How many bednets does your household possess? .... [__|__]

D10. At any time in the past 12 months, has anyone with a back-pack come and sprayed the interior walls of this house with insecticide to kill mosquitoes?
Read out options, only enter one answer .... [__]

1 = Yes; 2 = No; 3 = Don't know

DEWORMING USE

E1. Have you received treatment for worms in the last year?
Read out options, only enter one answer .... [__]

1 = Yes; 2 = No; 3 = Don't know

E2. If yes, where did you receive treatment?
Read out options, only enter one answer .... [__]

1 = School**;** 2 = Health centre; 3 = Home; 4 = Community programme; 5 = Shop; 6= Others *specify* [_____________________]

E3. How many tablets were you given?
Enter number .... [__|__|__]
Enter if 999, if Don't know

E4. What colour were the tablets?
Enter one answer .... [__]

1 = White; 2 = Yellow; 3 = Blue; 4 = Others **...***specify* [_________________]

ABSENTEEISM and RECENT ILLNESS

F1. During this school term, have you been absent from school due to illness?
Read out options, only enter one answer .... [__]
1 = Yes; 2 = No; 3 = Don't know

F2. In the last 2 weeks, have you been absent from school due to illness?
Read out options, only enter one answer .... [__]
1 = Yes; 2 = No; 3 = Don't know

F3. If absent in the last 2 weeks due to illness, what was illness ? **Read out options. Multiple options are allowed**

1= Headache .... [__]
2= Stomach ache .... [__]
3= Malaria .... [__]
4= Vomiting .... [__]
5= Diarrhoea .... [__]
6= Cough .... [__]
7= Eye infection .... [__]
8= Other *specify* [_____________________]

F4. Do you have fever or hot body today?
Read out options, only enter one answer .... [__]

1 = Yes; 2 = No; 3 = Don't know

F5. In the last 2 weeks, have you had fever or hot body**?**

Read out options, only enter one answer .... [__]

1 = Yes; 2 = No; 3 = Don't know

TRAVEL HISTORY

G1. During the last two months, have you made any overnight trips away from home ?

Read out options, only enter one answer ....[__]

1 = Yes; 2 = No; 3 = Don't know

G2. If yes to G1, how many trips did you make?

Enter number .... [__|__|__]

G3. We are now going to ask some questions about when and where you have travelled to in recent months.

Enter the following information

**Journey number**	Month of jour (numeric)	Number of days av	Districts	Town/market centre/village	Did you sleep ur a bednet?
Example	May [5]	7	Nandi	Kericho	No

Appendix 5: Teacher questionnaire

KENYA NATIONAL SCHOOL MALARIA SURVEY, 2009

TEACHER INFORMATION	
**Primary school code:** |_____|_____|_____|	**Primary school name:**
**District Code: [____]____]____]**	**District name:**
**Teacher ID |**_____|_____|_____|_____|_____|____|____|	**Date of visit:** |____|____|/|____|____|/|____|____| ***day month year***
**Teacher's last name**	**Teacher's first name**
**Age:** |_____|_____| **years**	**Gender:** ϶ **Male** ϶ **Female**

TEACHING EXPERIENCE

A1. What is your highest level of education?
Read out options, only enter one answer .... [___]

1 = Secondary incomplete; 2 = Secondary complete or above; 3 = University/college

A2. What teaching teacher have you received? .... [___]

1 = Teacher training college; 2=University

A3. How many years have you been teaching? .... [___[___]

A4. How long have you been teaching at this school? .... [___[___]

A5. What class do you now teach? .... [___]

KNOWLEDGE OF MALARIA

D1. What are the symptoms for malaria?
DO NOT READ OUT Please tick all mentioned:

1. Fever .... [__]
2. Weakness .... [__]
3. Vomiting .... [__]
4. Headache .... [__]
5. Diarrhoea .... [__]
6. Joint pains .... [__]
7. Malaise .... [__]
8. Poor appetite .... [__]
9. Chest pains .... [__]
10. Other (specify)[________________________________]

D2. What signs make you realize that fever in a child is serious?
DO NOT READ OUT Please tick all mentioned:

1. Sweating .... [__]
2. Shivering/chills .... [__]
3. Vomiting .... [__]
4. Diarrhoea .... [__]
5. Sleeplessness/restlessness .... [__]
6. Fever lasts more than 1 or 2 days ...... [__]
7. Convulsions .... [__]
8. Other (specify)[________________________________]

D3. In your opinion how can malaria be prevented?
DO NOT READ OUT Please tick all mentioned:

1. Use of insecticide treated mosquito net.................. [__]
2. Clearing bushes/stagnant water near dwellings...... [__]
3. Use of insecticides (Doom)...................................... [__]
4. Spraying of walls inside the house (IRS).................. [__]
5. Other (specify)[_____________________________________]

D4. Do you know the name of the antimalarial drug for uncomplicated malaria as recommended by the Ministry of Health?
DO NOT READ OUT Please tick all mentioned:

1. Artemether lumefantrine (AL)/ACT/Coartem .... [__]
2. SP/Fansidar .... [__]
3. Amodiaquine... [__]
4. Quinine.. [__]
5. Other (specify)[_____________________________________]

D5. Do you know where AL/Coartem/ACT can be obtained?
DO NOT READ OUT Please tick all mentioned:

1. Shop .... [__]
2. Government and mission health facility .... [__]
3. Pharmacy/Chemists .... [__]
4. Other (specify)[_____________________________________]

ABSENTEEISM and RECENT ILLNESS

C1. In the last 2 weeks, have you been absent from school due to illness?.. [___]

1 = Yes; 2 = No; 3 = Don't know

C2. In the last 2 weeks, have you been absent from school due to illness?... [__]

1 = Yes / ***Ndio***; 2 = No / ***La***; 3 = Don't know / ***Sijui***

C3. If absent in the last 2 weeks due to illness, what was illness ?.. [__]

1 = Headache / 2=Stomach ache/ 3=Malaria/ 4=Vomiting/ 5=Diarrhoea/ 6=Cough;
7 = Others *specify* [____]

C4. In the last 2 weeks, have you had fever or hot body**?** .... [__]

1 = Yes; 2 = No; 3 = Don't know

BEDNET USE and IRS

D1. Do you normally sleep under a bednet? .... [___]

1 = Yes; 2 = No; 3 = Don't know

D2. Did you sleep under a bed net last night?.. [___]

1 = Yes; 2 = No; 3 = Don't know

D3. What is the colour of your bed net .... [___]

1 = Blue; 2 = Green; 3 = White; 4 = Red; 5 = Others .... *specify* [___________________]

D4. When did you receive your bed net? .... [___]

1 = This school term; 2 = Last school term; 3 = Last school year***;*** 4=Before last school year

D5. From where did you receive your bednet? .... [___]

1 = School**;** 2 = Health cente; 3 = Home; 4 = Community programme; 5 = Others…… *specify* [______________________]
D6. Has the net ever been treated with an insecticide?.. [___]<br><br>1 = Yes; 2 = No; 3 = Don't know
D7. If yes in D6, where was the net treated?... [___]<br><br>1 = Pretreated; 2 = Treated at home; 3 = Don't know; 4 = Other Specify [____________________________________]
D8. If yes in D6, when was the net treated?.. [___]<br><br>1= Pretreated net; 2 = Within the last 6 months; 3 = Not within last 6 months; 4 = Don't know
D9. How many bednets does your household possess?.. [___|___]
D10. At any time in the past 12 months, has anyone with a back-pack come and sprayed the interior walls of this house with an insecticide to kill mosquitoes...[__]<br><br>1 = Yes; 2 = No; 3 = Don't know

Appendix 6: School questionnaire

KENYA NATIONAL SCHOOL MALARIA SURVEY, 2009

SCHOOL INFORMATION AND DEMOGRAPHICS

Date of visit: |___|___|/|___|___|/|___|___|
day *month* *year*

**District name**:	**District code:** |___|___|___|
**Name of school**:	**School code:** |___|___|___|
**GPS Longitude**: |___|___| : |___|___|___|___|___| E	**GPS Latitude**: |___|___| : |___|___|___|___|___| (N/S)<br>Negative ☐ Positive ☐ (tick as appropriate)
**School MoE code**:	**Start of school term:** |___|___|/|___|___|/|___|___|
**School type**: Day ☐ Boarding ☐ Boarding ☐	**Gender of pupils**: Mixed ☐ Boys ☐ Girls ☐
**Name of head teacher:**	**Head teacher phone number:**

**A. SCHOOL DEMOGRAPHICS**	**ECD**	**P1**	**P2**	**P3**	**P4**	**P5**	**P6**	**P7**
A1.Total boys enrolled:								
A2. Total girls enrolled:								
A3. Total boys present today:								
A4. Total girls present today:								
A5. Total male teachers:								
A6. Total female teachers:								

WATER and SANITATION FACILITIES

B1. Does the school have any of the following? Ask to see. ***Enter 1 =Yes and 2 = No***

Separate toilets for boys and girls .... [__]

Hand washing facilities near the toilets.. [__]

First Aid kit.. [__]

If yes, what does it contain? [________________________________]

B2. What is the main source of water for drinking for students in this school?
Only enter one answer .... [__]

1=Piped/tap water; 2=Borehole or well; 3=Rain water; 4=Stream or river; 5=Bought; 6=Bottled water;
7=Others.. *specify* [______________________]

B3. What type of sanitary facilities does the school have?
Only enter one answer .... [__]

1 = Water borne; 2 = VIP latrine; 3 = Ordinary latrine; 4 = Others (specify) [__________________________]

B4. If the school has hand washing facilities near the toilets, what type are they?
Only enter one answer ....[__]

1 = tap water; 2 = hand wash basin; 3 = leaky tins; 4 = Others (Specify) [____________________

B5. If the school has hand washing facilities near the toilets, is there soap?
Only enter one answer ....[__]

1 = Yes; 2 = No

B6. How many functional sanitary facilities does the school have?
Boys .... [____]
Girls .... [____]
Male teachers .... [____]
Female teachers .... [____]

B7. How many of those functional sanitary facilities are clean?
Boys .... [____]
Girls .... [____]
Male teachers .... [____]
Female teachers .... [____]

SCHOOL HEALTH ACTIVITIES and IEC MATERIAL

C1. In the last 12 months, has the school involved in any of the following school health activities? ***Enter 1 =Yes and 2 = No***

School feeding programme .... [__]

School deworming .... [__]

Water and sanitation programme .... [__]

HIV/sex education programme .... [__]

Malaria control activities .... [__]

Other, please specify [____]

Please provide details of the above programmes:

C2. Does the school have any of the following? ***Enter 1 =Yes and 2 = No***

Malaria IEC posters on display in the classrooms .... [__]

Malaria IEC posters on display in the headteachers office .... [__]

Malaria IEC booklets in the school library .... [__]

Other malaria IEC material, please specify [____________________________________]

Please provide further details of the above:

Appendix 7: Survey information sheet

KEMRI-Wellcome Research Programme Information Sheet

Kenya National School Malaria Survey

Introduction
We are staff from the Kenya Institute for Medical Research (KEMRI) and the Ministry of Health, and are conducting research in primary schools on the prevalence of malaria and anaemia and the use of mosquito nets among school children. In selected schools, we will also collect information on the occurrence of worms. As teachers and parents of these children, we need your permission and co-operation.

What is Research?
KEMRI is a Government organization that carries out health research to learn more about diseases that affect children and adults in Kenya. Sometimes research involves investigating the health of individuals and the diseases which affect them. This is the type of research we are here to do today.

What is this research about?
In this research, we want to learn more about the occurrence of malaria and anaemia among school children in Kenya. We are visiting a total of 533 primary schools in all the provinces in Kenya and in each school, will be randomly selecting one hundred and ten children.

What will it involve for my child(ren)?
We will take a small sample of blood from your child(ren) to find out if they have malaria parasites and to determine their haemoglobin level. Haemoglobin gives blood its red colour and carries oxygen around the body. If there is not enough haemoglobin a child can become easily tired – this is called anaemia. We will use a sterile needle to make a prick on a finger and collect one drop of blood and measure the amount of haemoglobin in a machine. This sample will be less than half a tsp (2ml) and will be taken from your child(ren)'s finger. These samples will be used to prepare slides which will be examined under a microscope. In addition, in a sub-set of schools we will also take stool samples to test for intestinal worms

Are there any disadvantages involved in taking part?
There are minimal risks with participating in this study. The finger prick blood sample may cause minor temporary discomfort for children. The provision of stool samples may cause minor embarrassment.

Are there any benefits to me/my child of taking part?
Children found to be infected will be provided, free of charge, appropriate treatment. The drugs used for treatment in this study are known to be safe in most people, and are in common use in Kenya. These drugs are government recommended treatment for malaria - Artemisinin-based combination therapies (ACTs) - and albendazole treatment for intestinal worms. Those children suspected to have severe anaemia will be referred to the local health centre for appropriate treatment. The information generated from the survey will be very useful for making decisions about parasite control in your community and our country as a whole.

What will happen if I don't agree to participate?
All participation in research is voluntary. You are free to decide if you want your child(ren) to take part or not. Your child(ren) will still receive the recommended standard of care for worms if they do not take part. If you do agree you can change your mind at any time and withdraw your child from the research. This will not affect their care now or in the future.

What happens to the samples?
All of the tests that are needed as part of this research will be done locally in Kenya.

Who will have access to information about me/my child in this research
We will take strict precautions to safeguard your child's personal information throughout the study. All our research records are stored securely in locked cabinets and password protected computers. Only a few people who are closely concerned with the research will be able to view information from participants.

Who has allowed this research to take place?
All research at KEMRI is approved by national independent expert committees in Nairobi and a committee in Kilifi to make sure the research is conducted properly and that participants' safety and rights are respected.

What if I have any questions?
You may ask any of our staff questions at any time. You can also contact those who are responsible for the care of your child and this research: Dr. Simon Brooker, at KEMRI – Wellcome Trust, P.O. Box 43640 - 00100, Nairobi, Kenya. Tel: +254 20 2715160 or 2720163 or 2719936, Mobile: +254 724 350 056

If you have questions about your rights as a study participant, concerns about the research or if you want to ask someone independent anything about this research please contact: Community liaison officer, at KEMRI – Wellcome Trust, P.O.Box 230, Kilifi. Telephone: 041 522 063 ***Or*** Chair, KEMRI/National Ethics Review Committee. P. O. BOX 54840-00200, Nairobi, Telephone: 020 272 2541

Appendix 8: Malaria blood slide preparation

1. Purpose / introduction:
   To provide guidelines for the proper preparation of high quality, standardized thick and thin malaria blood smears for microscopic examination of malaria parasites.

2. Specimen:
   Capillary blood drawn directly from a finger prick or heel

3. Equipment / materials/ reagents:
   - Grease free glass slides / Precleaned slides
   - Slide box
   - Slide folder
   - Labels / Pencils
   - Alcohol swabs/Alcohol pads
   - Dry swabs
   - Lancets
   - Gloves
   - Biohazard container
   - Sharps containers
   - Bins for contaminated materials
   - Smear preparation template

NB: Always wear protective gadgets before performing any procedures.

4. Method:

Labeling the slides
- Select a new glass slide free from dust particles and grease.
- Handling by the edges, label the slide in pencil on the frosted portion of the slide with the project name, child ID and the date of the sample collection.

For example:

KNMS<br>60101001<br>07/09/09	

- **The child ID on the slide must match with the child ID on the child information form**.
- **ALL slides must be correctly labeled as shown above**.

Preparation of the child
- Ensure the child is relaxed.
- Explain to the child what you intend to do before any sample collection and get consent before the finger Prick.

5. Preparation of blood smears

- Clean the ring finger with cotton swab soaked in spirit. Use firm strokes to remove grease, perspiration, or dirt that may be on the skin.
- Dry the finger with dry cotton gauze.
- Puncture the ball of the finger with a sterile lancet. **DO NOT REUSE LANCETS.**
- Wipe out the first drop of blood and apply gentle pressure on the finger to release a free flowing drop of blood.
- Collect samples for both thick and thin malaria blood films on the same side of a single slide ensuring that the blood is not placed on the frosted portion of the side of the slide.
- For the thick smear, place the drop of blood from the finger prick by touching the drop slightly on the slide, controlling the amount of blood to be collected (approx. 12 μl of blood).
- Using a circular motion with a slide, Spread the blood to form a larger circle. Avoid air bubbles.
- For the thin smear, place a drop of blood from the finger prick by touching the drop slightly on the slide to acquire approximately 4μ l of blood near the center of the slide.
- Using a slide with a smooth edge, place it in front of the drop of blood. Pull back the slide and hold until the blood is evenly spread along the width of the spreader slide.
- Raise the slide to an angle of 30 to 45 and spread at a moderate speed in a continuous motion in order to create a feathered edge of the thin smear before reaching the other end of the slide.
- Protect the smear from flies and dust by placing the smear in slide folder.
- Place the slide flat on the rack and let the films dry horizontally.
- **The dried films should be stained within 72 hours to avoid autofixation**.

Appendix 9: Staining of blood slides

1. Equipment
   - Giemsa stock solution
   - Buffered water to pH 7.2
   - Methanol
   - Timer
   - Staining jars
   - Slide drying rack
   - Measuring cylinder (500ml)
   - Measuring cylinder (20ml)
   - Beaker

2. Methods
   - Fix the thin blood film by dipping it in a container of methanol for a few seconds making sure that the thick film is not in contact with the methanol.
   - Prepare 3% giemsa stain following the dilutions shown in the table with a final volume depending on how many slides are expected to be stained on that day. Fresh Giemsa stain should be prepared daily from the stock solution (100% Giemsa). Thick and thin smears are stained with 3% Giemsa for 45 minutes.

Staining solution	Volume of stock solution	Final volume achieved by adding buffered water
**3% Giemsa stain** to be used for thin and thick smears	1.5 ml	50ml
	3 ml	100ml
	6 ml	200ml

1. Place the slides into the staining jars with the thick films together on one side
2. Pour the 3% Giemsa in the staining jars (do not pour directly on the thick films) OR pour onto the slides to cover the side to be stained.
3. Set the timer and wait for 45 minutes.
4. Gently pour clean water into the staining jar to float off the “scum”. Rinse the slides with distilled water.
5. Place the slides on the wooden drying rack until to dry.
6. Pack the slides into slide boxes when dry, checking that each slide has child ID clearly marked.
7. Label slide boxes clearly on the outside with the school, ID number range and date.

Appendix 10: Collecting filter paper blood spots for the recovery of human antibodies

Kindly provided by Dr Chris Drakeley, London School of Hygiene and Tropical Medicine, Jan 2008
(Comments, queries & questions: chris.drakeley@lshtm.ac.uk)

Background
Collecting blood spots onto filter paper is a well-established method for storing blood for biochemical screening assays[1-3] and more recently has been used for antibody assays[4,5]. Although the stability of analytes in blood spots has been extensively studied[2] and guidelines produced[3] for blood spot collection, transport and storage, the stability of antibodies within dried blood spots has only been studied in small-scale studies (e.g.[4,5]). The relationship between blood spot size and blood volume not quite linear with a slight tendency for the centre of the spot to be more concentrated than the periphery, as noted before[6]. Antibody recovery did not differ significantly between discs punched from the centre and periphery of multiple blood spots. Approximating this relationship as a straight line gave a spot density of 3.6±0.3 $mm^2/\mu l$, thus <u>a disc of 2.6 m diameter corresponds to an original blood volume of 1.5 µl.</u>

References 1. Guthrie R. (1992) Screening :5-15. **2.** Davis G, Poholek R (1979) Clin Chem. 25:24-5. **3.** Hannon WH, et al. National Committee for Clinical Laboratory Standards; 1997 NCCLS Document LA4-A3. **4.** Thaver S, Draper CC. (1974) Trans R Soc Trop Med Hyg.68 8. **5.** McDade TW *et al.*(2000) Psychosom Med. 62 560-7. 6.Mei JV *et al*. (2001) J Nutr. 131 1631S-6S.

A. Preparation of filter papers

1. Cut Whatman 3M filter papers into strips of approximately 2cm x 5cm (figure 1)

Fig 1

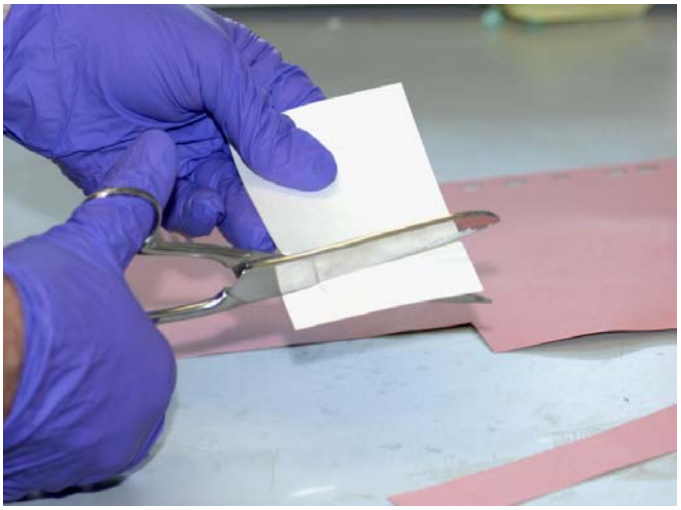

2. Cut a piece of card twice the length of the filter paper (figure 2) and staple the filter paper inside (figure 3)

Fig2

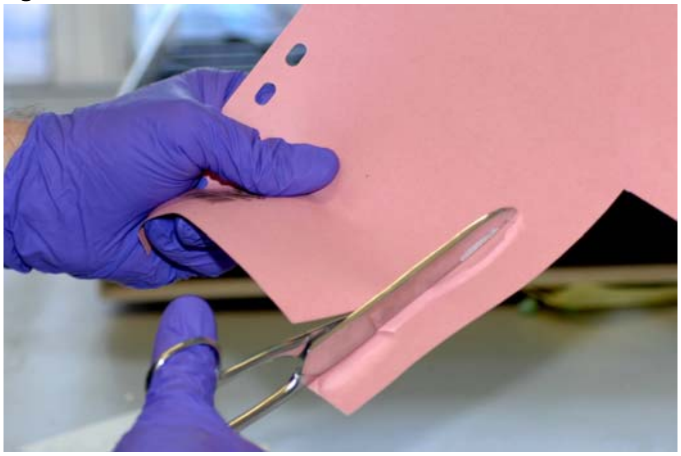

Fig 3

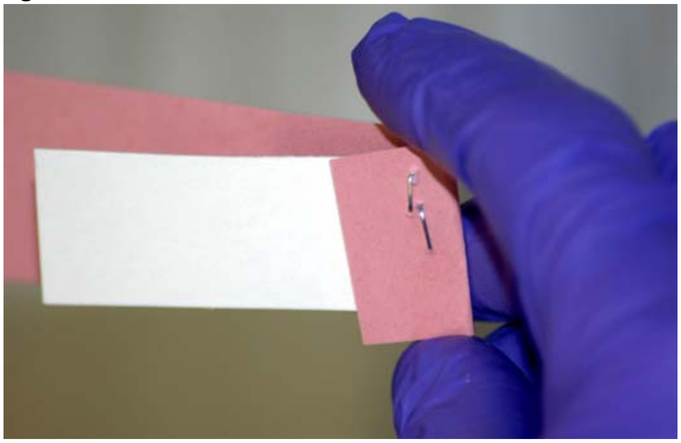

3. Fold the card round the filter paper to protect it (figure 4). Make as many filter papers as required

Fig 4

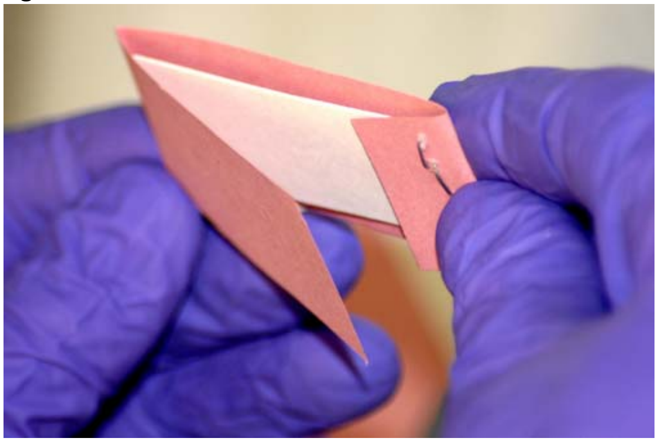

B. Making blood spots

1. Blood is taken via finger/heal prick

2. Place tip of finger or heal on filter paper and allow blood to move into filter paper by capillary motion (figure 5)

Fig 5

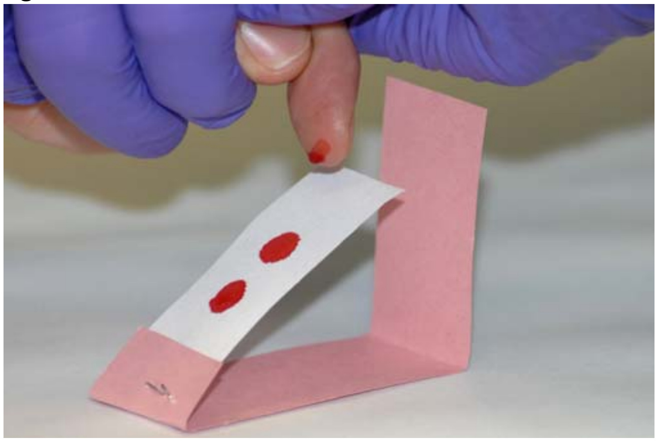

3. 3 spots are made on the filter paper (figure 6)

Fig 6

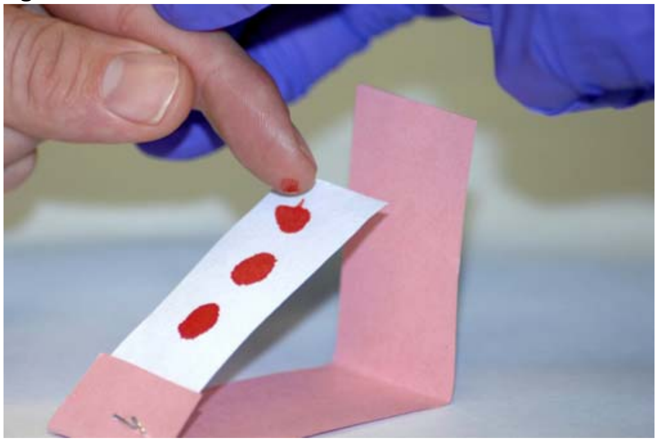

4. Label the filter paper with the appropriate information, the child ID, survey date and the project name. (figure 7)

Fig 7

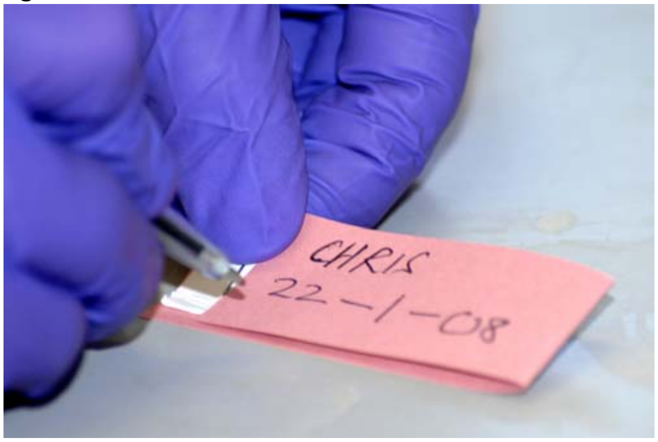

5. Allow the spots to air dry for 20-30 minutes

C. Storage of blood spots

1. Filter paper cards are stored in boxes made from the same card. Filter paper cards are stored back to back in the boxes (figure 8 & 9)

Fig 8

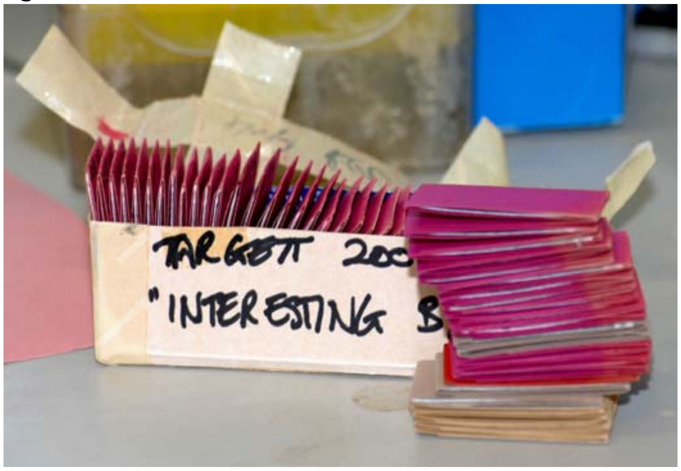

Fig 9

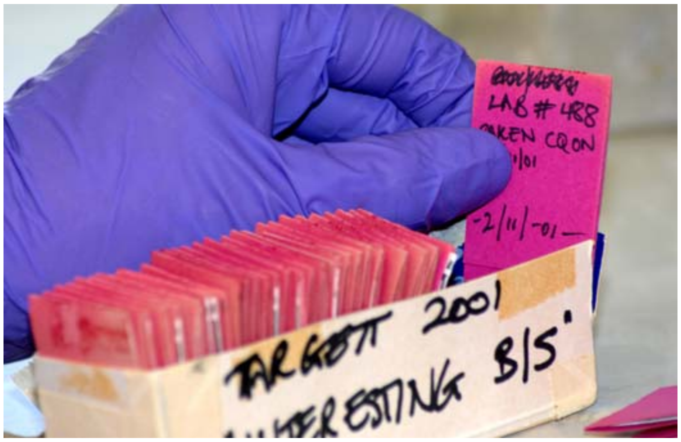

2. boxes are stored together (figure 10) with dessicant (figure 11) in large air tight plastic boxes (figure 12)

Fig 10

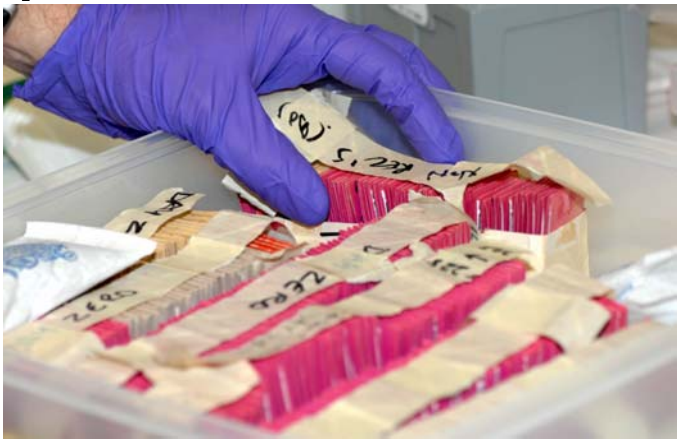

Fig 11

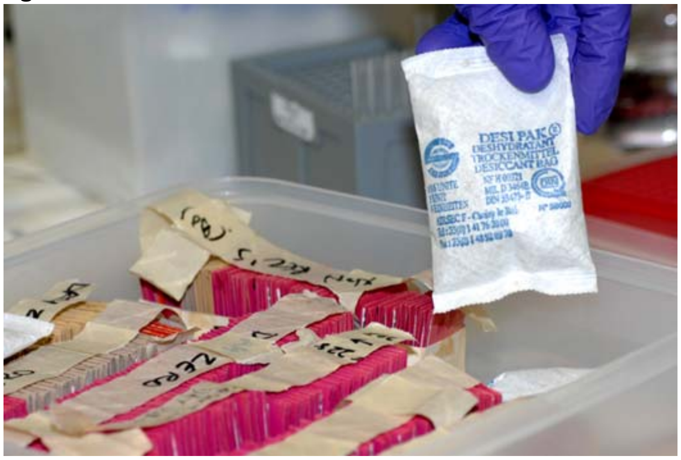

Fig 12

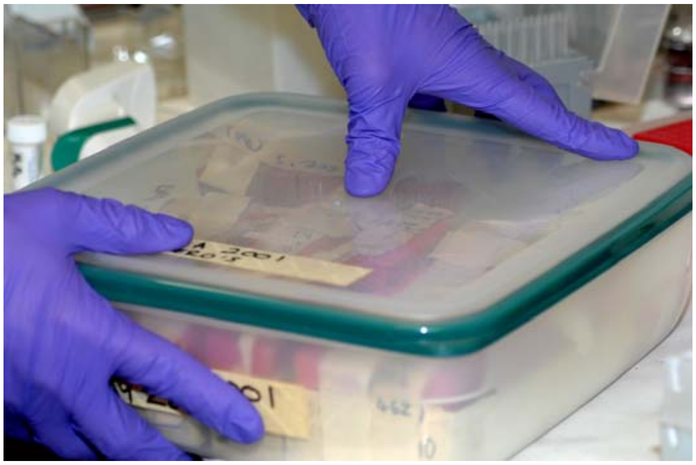

3. Boxes are stored in the fridge (figure 13)

Fig 13

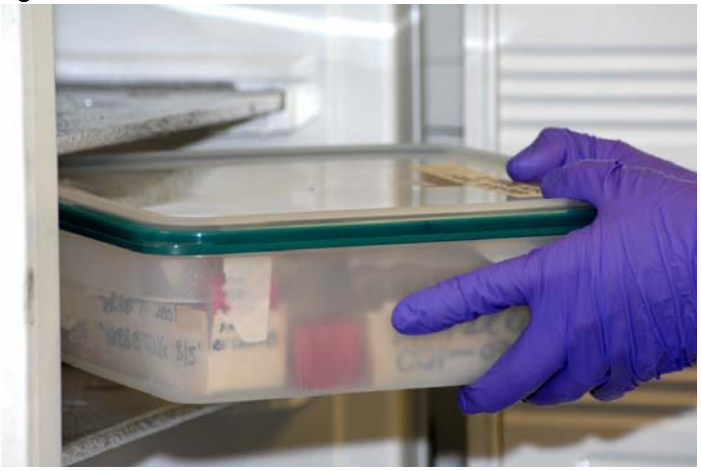

4. Dessicant should be checked and changed regularly (minimum once per month)

5. For longer term storage individual filter papers or groups of filter papers can be stored in self sealing plastic bags to minimise any moisture getting on the filter paper.

Appendix 11: Haemoglobin assessment using HemoCue device

Purpose: To describe the method for operating the HemoCue device and correctly obtaining haemoglobin readings from finger-prick blood samples.

Background / Rationale: Haemoglobin levels can be influenced by malaria or infection with helminths such as hookworm. Chronic anaemia has an adverse effect on the health and development of children, and this information is integral to this study. The correct method of testing blood for haemoglobin levels should be followed in order that results are comparable.

Supplies and materials:
- HemoCue device
- New, clean, HemoCue microcuvette

Procedure:
1. Perform finger prick as in appendix 3.
2. Collect blood drop in microcuvette in one continuous process.
3. Wipe off excess blood on the outside of the microcuvette.
4. Check there are no bubbles in the microcuvette. Small bubbles at the edge can be ignored.
5. Place in the cuvette holder and push the tray closed.
6. After 15-60 seconds the reading will be displayed. Record this on child information sheet.
   Note: The haemoglobin reading should be recorded on the child information form in **g/L.**
7. Open cuvette holder and discard the microcuvette.

Appendix 12: Preparation of the malaria rapid diagnostic test (RDT)

Purpose: To provide guidelines on RDT preparation and interpretation.

Equipment / materials/ reagents:

- Paracheck devices
- Labels / Pencils
- Alcohol swabs/Alcohol pads
- Dry swabs
- Industrial methylated spirit 70% $^{v}/_{v}$
- Lancets
- Gloves
- Biohazard container
- Sharps containers
- Bins for contaminated materials

Procedure

- Check the expiry date on the packet of the RDT to ensure it is still valid.
- Open the packaging to retrieve the RDT device, applicator and desiccant.
- Check if the desiccant in the pack is still blue, **if the desiccant is pink or colourless, the RDT device should not be used.** All the invalid and spoilt RDTs should be given to the team leader and brought back to Nairobi.
- Place the RDT device on a flat surface and clearly write the child ID on it.
- Tighten the vial cap of the clearing buffer provided with the kits in the clockwise direction to pierce the dropper bottle nozzle.
- Perform finger prick as in appendix 3, touch the sample applicator to the blood on the finger prick. Ensure that a loop of blood is retrieved and blot on the sample pad in sample well A.
- Add 6 drops of the clearing buffer into well B by holding the buffer bottle vertically.
- Write the time that blood and buffer were added on the well and start the timer.
- At the end of 15 minutes, read the results as follows;

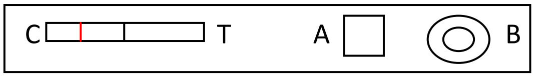

Negative for *P. falciparum*: only one pink coloured band.

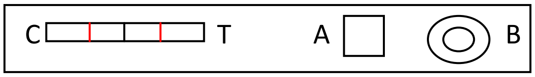

Positive for P. falciparum: In addition to the control band, a distinct pink coloured also appears in the test.

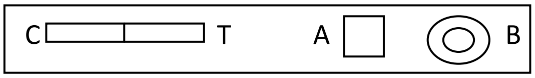

Invalid: The test should be considered invalid if no band appears on the device.

Note: Results should not be read after 15 minutes.

- Dispose of all sharps and blood contaminated items in sharps containers and biohazard containers, respectively.

- For children found to have *P. falciparum* infection, treat with Coartem according to guidelines in appendix 14.

Appendix 13: Kato-Katz preparation

1. *Purpose / introduction:*

To provide guidelines for the proper preparation of high quality, standardized Kato-Katz cellophane faecal thick smear technique for microscopic examination of nematode and *Schistosoma mansoi* ova

2. *Scope / responsibility:*

This SOP is applicable to all Laboratory technologists, Clinical officers, Nurses and field workers working in KEMRI / Wellcome Trust.

3. *Definitions:*

SOP: standard operating procedures

4. *Specimen:*

Stool sample provided by patient in a polypot.

5. *Equipment / materials/ reagents:*

- Grease free glass slides / Precleaned slides
- Slide carriers
- Labels / Pencils
- Wooden applicator stick
- Screen, stainless steel, nylon or plastic 60-105 mesh.
- Plastic template
- Plastic spatula
- Toilet paper or absorbent tissue.
- Newspaper.
- Hydrophilic cellophane, 40-50 µm thick, strips 25 x 30 mm in size
- Forceps

6. *Methodology:*

labelling the slides

- Select a glass slide free from dust particles and grease, preferably a new slide. If greasy clean with a soft tissue paper soaked in 70% IMS
- Label the slide in pencil on the frosted portion of the slide according to the study protocol involved.
- Slide labelling must match with the information on the accompanying request form.

Preparation of kato-katz slides

- Place a small (corn seed size) mound of faecal material on newspaper or scrap paper and press the small screen on top so that some of the faeces are sieved through the screen and accumulate on top.

- Scrape the flat-sided spatula across the upper surface of the screen to collect the sieved faeces.
- Place template with hole on the centre of a microscope slide and add faeces from the spatula so that the hole is completely filled. Using the side of the spatula pass over the template to remove excess faeces from the edge of the hole (the spatula and screen are to be carefully washed and reused).
- Remove the template carefully so that the cylinder of faeces is left on the slide.
- Cover the faecal material with the pre-soaked cellophane strip. The strip must be very wet if the faeces are dry and less so if the faeces are soft (if excess glycerol solution is present on upper surface of cellophane wipe with toilet paper).
- Invert the microscope slide and firmly press the faecal sample against the hydrophilic cellophane strip on another microscope slide or on a smooth hard surface such as a piece of tile or a flat stone. The faecal material will be spread evenly between the microscope slide and the cellophane strip.
- Carefully remove slide by gently sliding it sideways to avoid separating the cellophane strip or lifting it off. Place the slide on the bench with the cellophane upwards. Newspaper print can be read through the smear after clarification. Water evaporates while glycerol clears the faeces.
- After ten minutes place slide onto microscope and count all eggs present on laboratory form.

Appendix 14: Urine filtration preparation

1. *Purpose / introduction:*
   To provide guidelines for the proper preparation of high quality, standardized urine filtration technique for microscopic examination of *Schistosoma haematobium* ova

2. Scope / responsibility:
   This SOP is applicable to all Laboratory technologists, Clinical officers, Nurses and field workers working in KEMRI / Wellcome Trust.

3. *Definitions:*
   SOP: standard operating procedures

4. *Specimen:*
   Urine sample provided by patient in a plastic specimen pot.

5. *Equipment / materials/ reagents:*

   - Grease free glass slides / Precleaned slides
   - Slide carriers
   - Labels / Pencils
   - 10 mL syringe
   - Swinnex filters
   - Swinnex filter holder
   - Coverslip

6. *Methodology:*
   Labelling the slides
   - Select a glass slide free from dust particles and grease, preferably a new slide. If greasy clean with a soft tissue paper soaked in 70% IMS
   - Label the slide in pencil on the frosted portion of the slide according to the study protocol involved.
   - Slide labelling must match with the information on the accompanying request form.

   Urine collection
   - It is preferable to obtain total urine collected over the time period between 10.00h and 14.00h as it has been shown that a maximum concentration of eggs are excreted at this time.
   - If the urine cannot be examined within an hour of collection, it is advisable to add 1mL of undiluted formalin to preserve any eggs that may be present.

- If processing is delayed, refrigeration is preferable to storage at ambient temperature.

Preparation of slides

- Draw 10 mL urine into a syringe, and then connect to a Swinnex filter (pore size 12 µm).
- Gently ease the urine through the filter.
- Draw in 20 mL of air and ease this through the filter.
- Remove the top of the filter and place the membrane on to a microscope slide.
- Add a drop of saline, apply a coverslip and view microscopically using a low power objective.

Appendix 15: Weight dosage charts

Artemether-lumefantrine (Coartem; Novartis) administered twice daily for three days as tablets containing 20 mg of artemether plus 120 mg of lumefantrine in a fixed dose combination at a dosage.

Weight (kg)	Age	Day 1		Day 2		Day 3	
		Morning	Evening	Morning	Evening	Morning	Evening
5–14	3mo–2yrs	1	1	1	1	1	1
15–24	3–7 yrs	2	2	2	2	2	2
25–34	8–10 yrs	3	3	3	3	3	3
> 35	10+ yrs	4	4	4	4	4	4

Albendazole: 400mg tablet. For treatment of *Ascaris lumbricoides Trichuris trichiura* and hookworm species infections. Single dose treatment

Age	Dose
Under 1 year	Do not treat
1 – 2 years	½ tablet
Children over 2 years	1 tablet
Adults	1 tablet

Praziquantel: 600mg tablet. For treatment of infection with *Schistosoma mansoni.* Single dose treatment. The standard dose is 40mg/kg body weight. Height can also be used to determine dose required according to the WHO praziquantel dose pole.
Children under 5 years of age are unlikely to be infected with *S. mansoni*, but praziquantel is safe for use children aged 1 to 5 years.

Weight (kg)	Height (cm)	Number of 600mg tablets
15 – 22.5	94 – 110	1
22.5 – 30	110 – 125	1 ½
30 – 37.5	125 – 138	2
37.5 – 45	138 – 150	2 ½
45 – 60	150 – 160	3
60 – 75	160 - 178	4
> 75	> 178	5

Appendix 16: Data entry on Ms Access database

1. Guidelines for laptop care and maintenance

It is recommended that you follow certain guidelines to prevent possible damage to your HP iPAQ and to keep it in a good condition.

Using your laptop

- Avoid using your the laptop in dusty and extremely high or low temperature environments.
- Do not spill water on the laptop or expose it to the rain.
- Avoid removing the battery without turning off the laptop.
- Do not apply strong force or shocks to the laptop.
- Keep the laptop and its accessories away from children.
- Do not insert metal objects into the charging/communications port of the laptop. This can short-circuit the battery and result in danger.
- Always turn off the laptop when your are through done using it to avoid wastage of battery life.

Carrying or storing your laptop

- Do not leave the laptop in extremely high or low temperature environments.

Using accessories

- Use original laptop batteries and chargers to charge the laptop.

2. Using the KNSS database

To open the database, double click on the shortcut icon on the desktop. If there is no shortcut, then open the C drive or whatever other drive in the laptop and open the KNSS folder then double-click on the database called KNSS to open.

The following screen should appear.

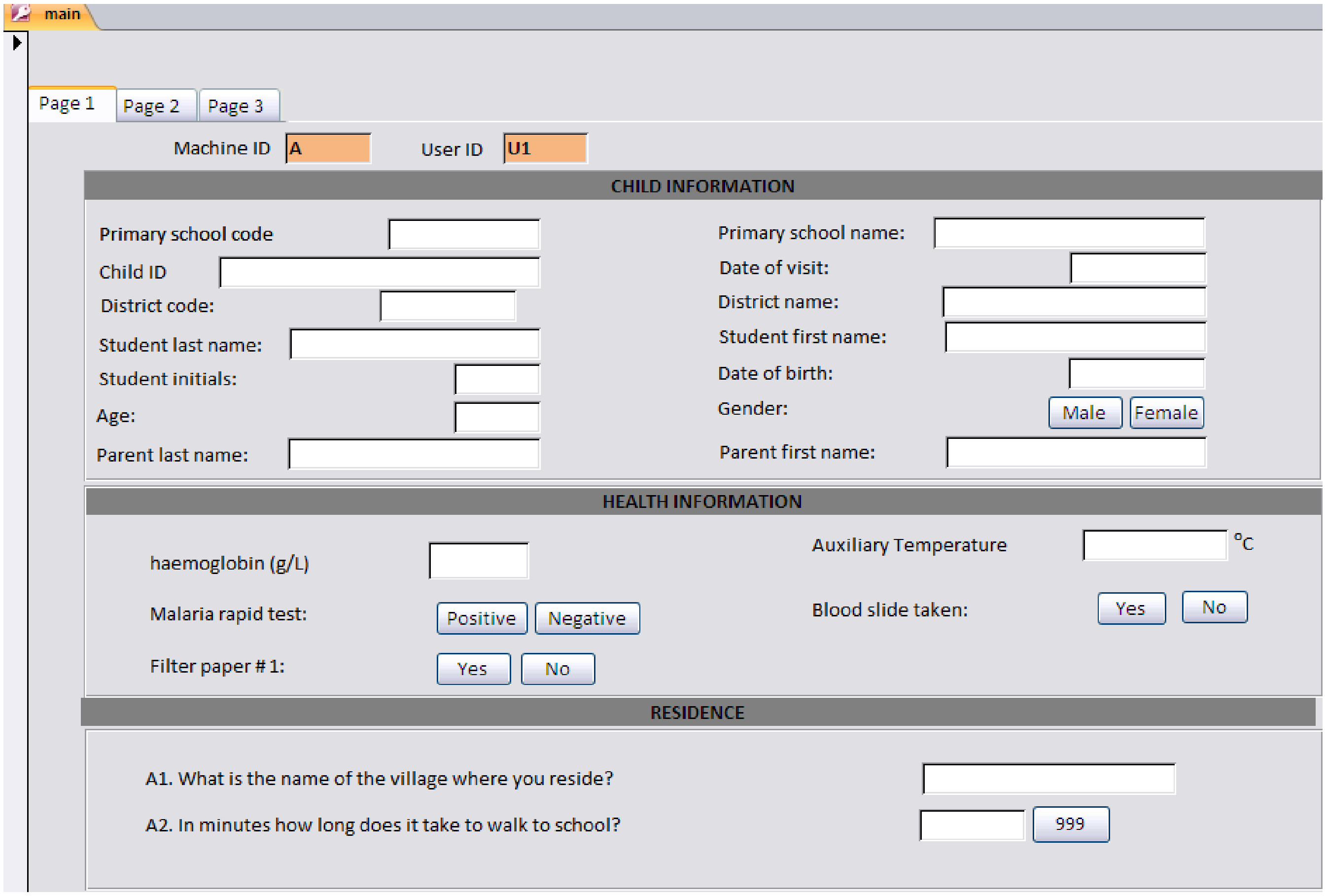

Adding a new record

To start entering data, fill in the details required starting with page 1 up to page 3.

To start a new record, click on the arrow with a star at the bottom of the page as shown in the figure below:

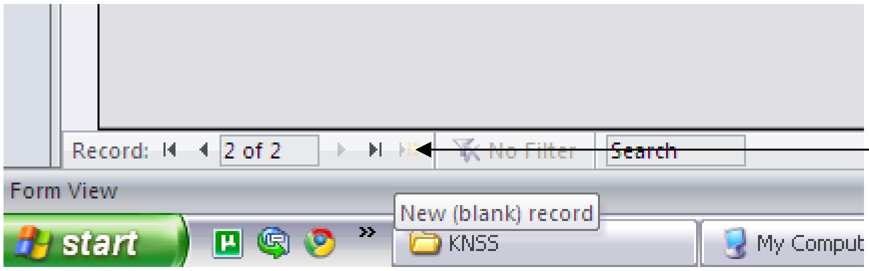

Click on the shown arrow to create a new blank record

Field types:

1. Text box

This is a field in which you are required to type in text, numeric values or a combination of both. The amount of text or numbers to be entered may be limited depending on the question type. E.g.

For:

- child id, only numeric values can be entered
- Primary school name, any text can be entered here.

2. Option group buttons

These are fields that only allow one to select **ONLY ONE** option by clicking on a button. Once one option is selected it is highlighted and the other option is deselected. E.g.

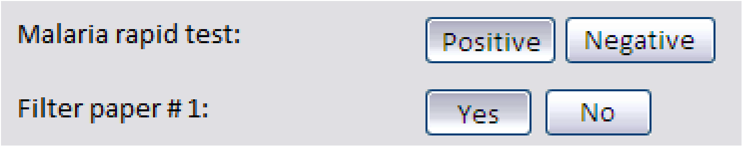

In the above example, only one option is selected **positive** for the first question and **yes** for the second question.

3. Combo box

This field contains a drop down list box from which you select only one option. Click on the arrow on the right to view the list of items available then click the item you would like to select. To select on another item, simply select it from the list again.

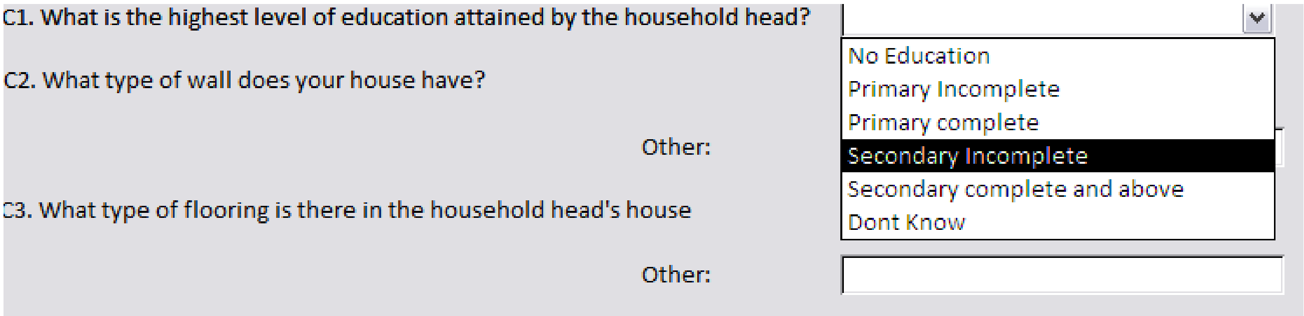

4. Check box

This field contains a set of options of which you can select one or more options. Simply click on the small box next to the label in order to tick the required option as shown in the figure:

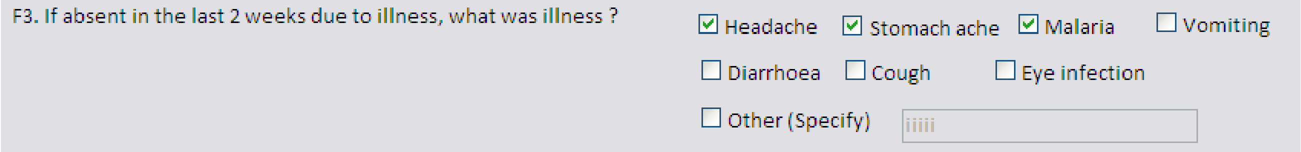

5. Sub form

This is also called a form within a form. Click on the sub form to enter data as you would in the main form. An example of a sub form is shown below:

Fill in information on the each journey in a new line that appears below the first journey in the sub form.

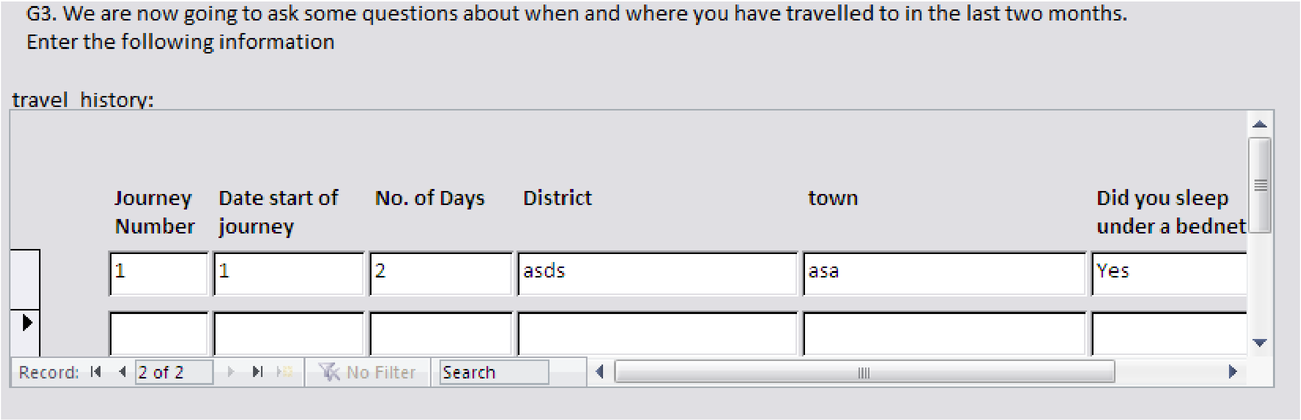

Skip logic

Answers to some questions may enable or disable other questions. An example is shown below;

This is illustrated in the following series of images

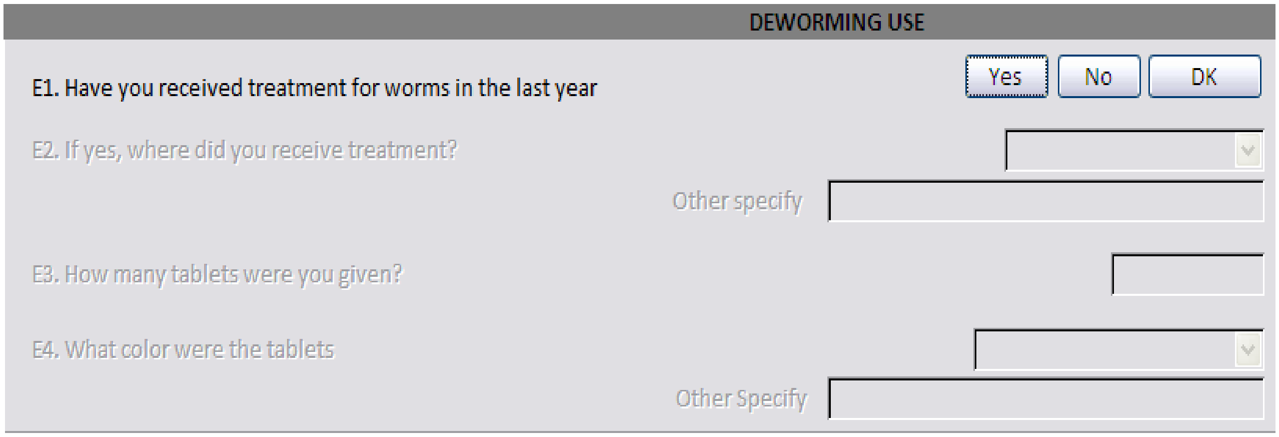

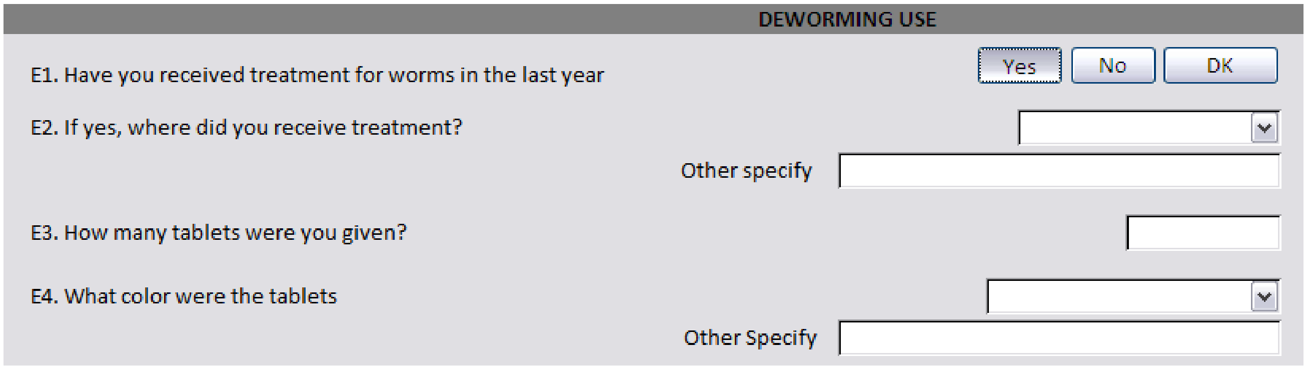

Before any answer is selected, the fields E2 to E4 are disabled. Hence question E1 has to be answered first. yes is selected, then the subsequent set of fields becomes enabled as shown below.

Merging data from several computers:

3. Merging data from several computers

Before starting the merge, insert the flash disk containing the folder KNSS\MERGE\KNSS_merge.mdb

When merging the databases from different tables to the flash disk for backup use the following procedure:
1. While opening the database on the laptop, hold down the shift button while opening the database. (Hold down the button until the database opens then release)
2. Click on the navigation button on the left hand side to view the navigation pane as shown below

Click here to show the navigation pane

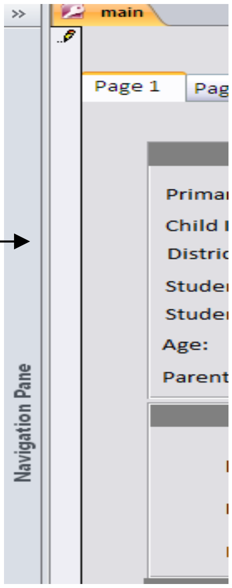

Navigation pane

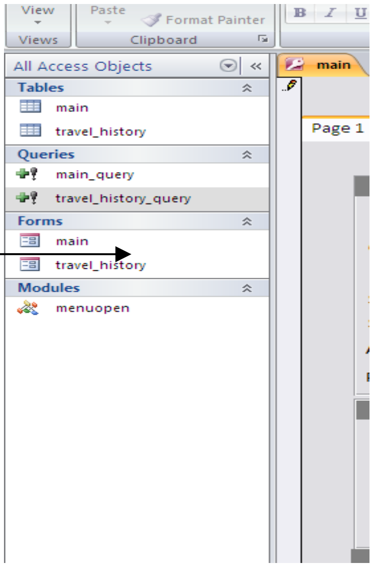

3. Double click on the main query and the following screen should appear

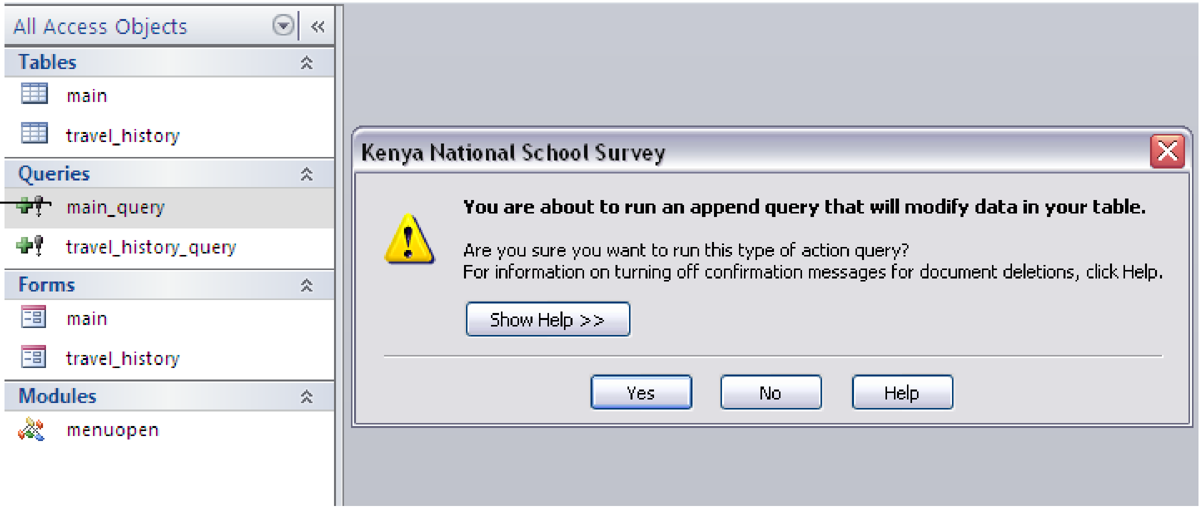

Main query

Click the Yes button.

The following screen should appear

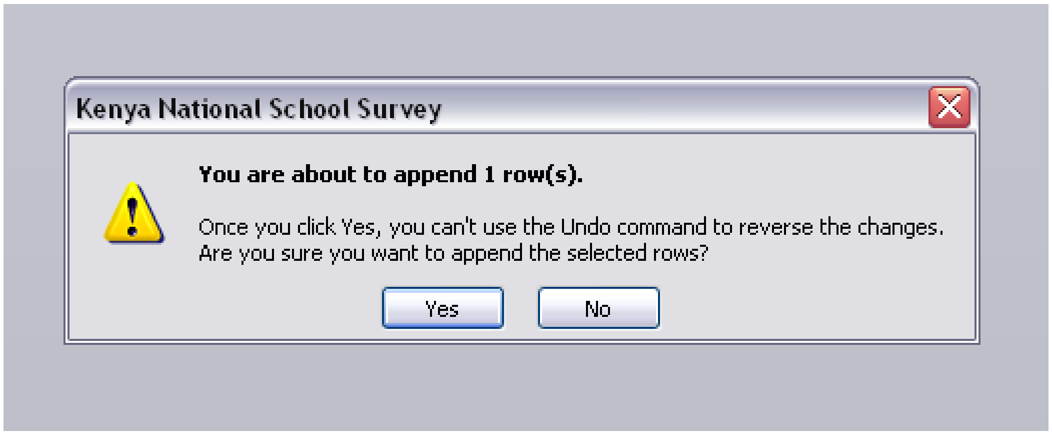

Click the Yes button to merge the data in the flash disk.

4. Exporting merged data

Open the flash disk containing the merged data in the path KNSS\MERGE\
The database containing the merged data is called KNSS_merge.mdb
Double click on KNSS_merge.mdb while holding down the shift key.
You should see the following navigation pane and access objects:

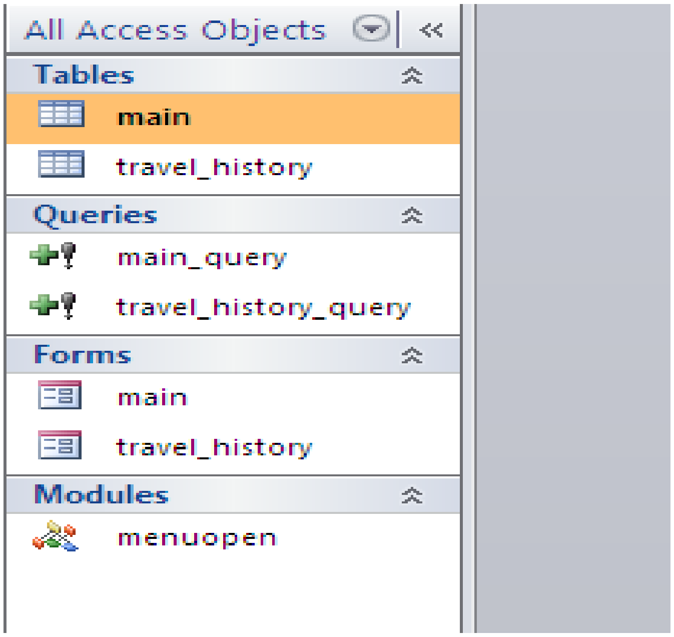

Right click on the main table ---export ---- excel to export the data to excel format

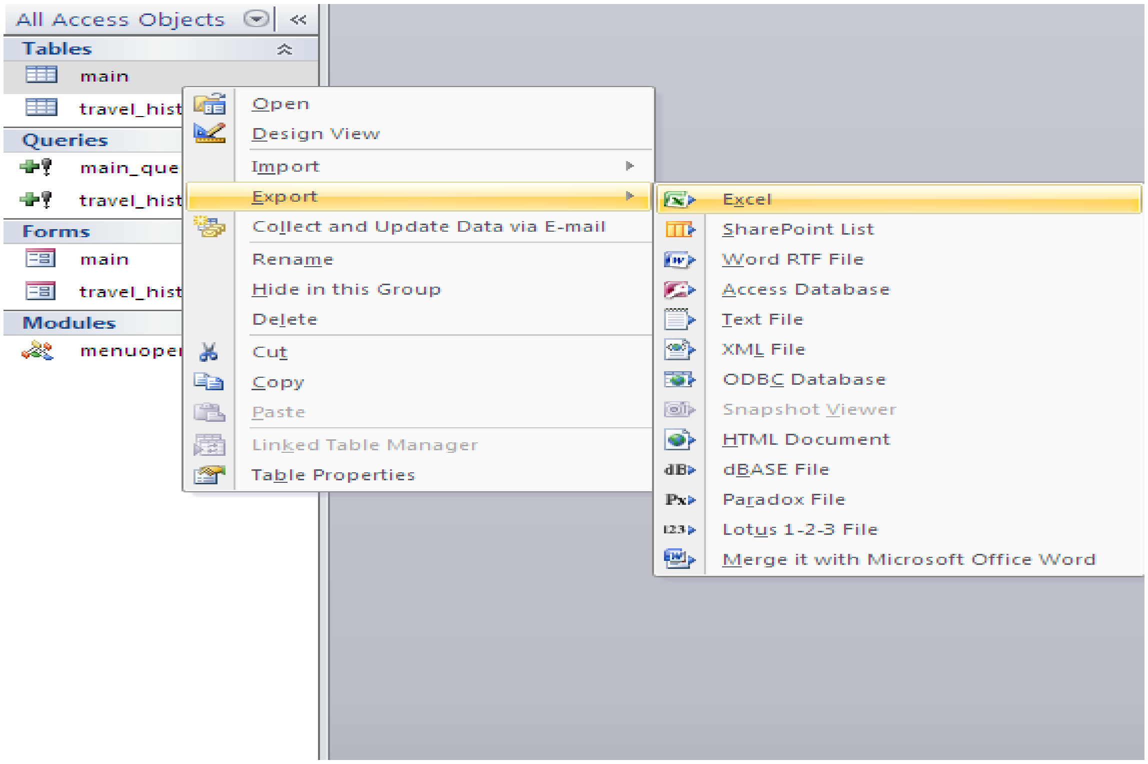

Click on browse to select a location to save the file

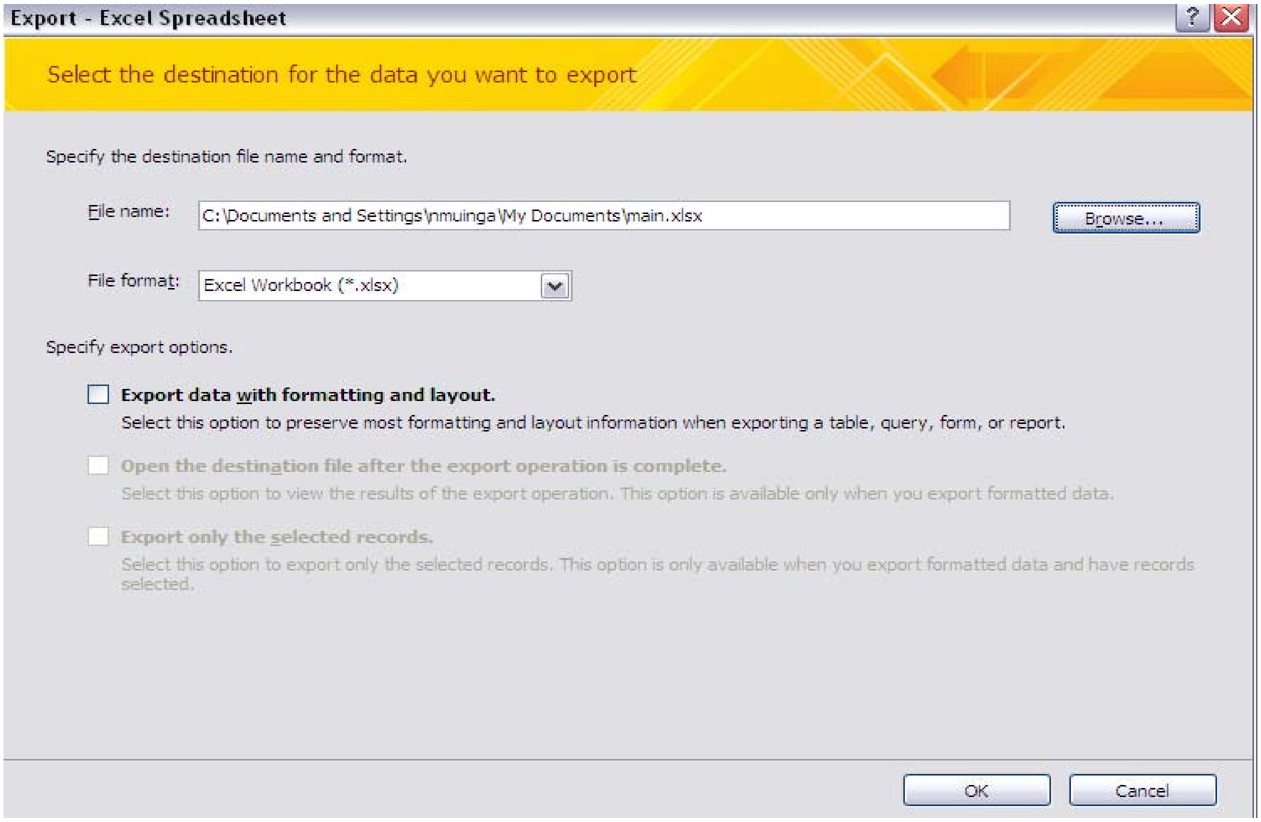

Type in the file name and select the format as excel 2003 as shown below

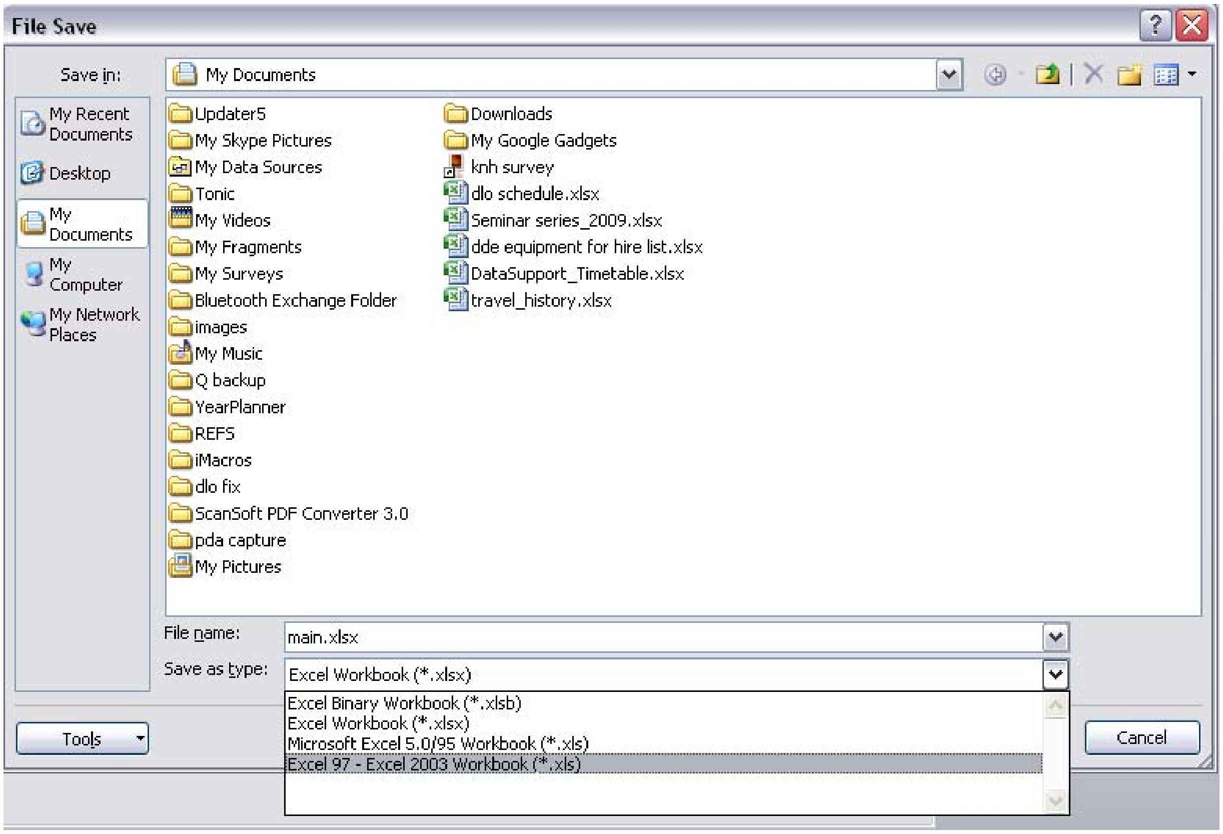

Repeat the same procedure for the travel history table.

The created created should be sent nightly via email as attachments to Nairobi.

Appendix 17: Definitions and Abbreviations

Definitions

Bed net	Net used (usually at night) to prevent mosquito bites
Indoor Residual Spraying	Refers to the long lasting insecticide and not aerosol sprays (e.g. Doom which is an aerosol sprays)
Siblings	A brother or sister from the same mother
Household	A person or a group of people living in the together in the same house, who share the same housekeeping arrangements
Household head	The person who is the head of the household

Abbreviations

Hb	Haemoglobin
RDT	Rapid diagnostic test
IRS	Indoor residual spray
DOB	Date of birth
