# Supplementary figures and images for: Implementing school malaria surveys in Kenya: towards a national surveillance system
Source: Malar J. 2010 Oct 30;9:306. doi: 10.1186/1475-2875-9-306 (PMC2984573; doi:10.1186/1475-2875-9-306)

**Additional file 2.** Microscopy results flowchart

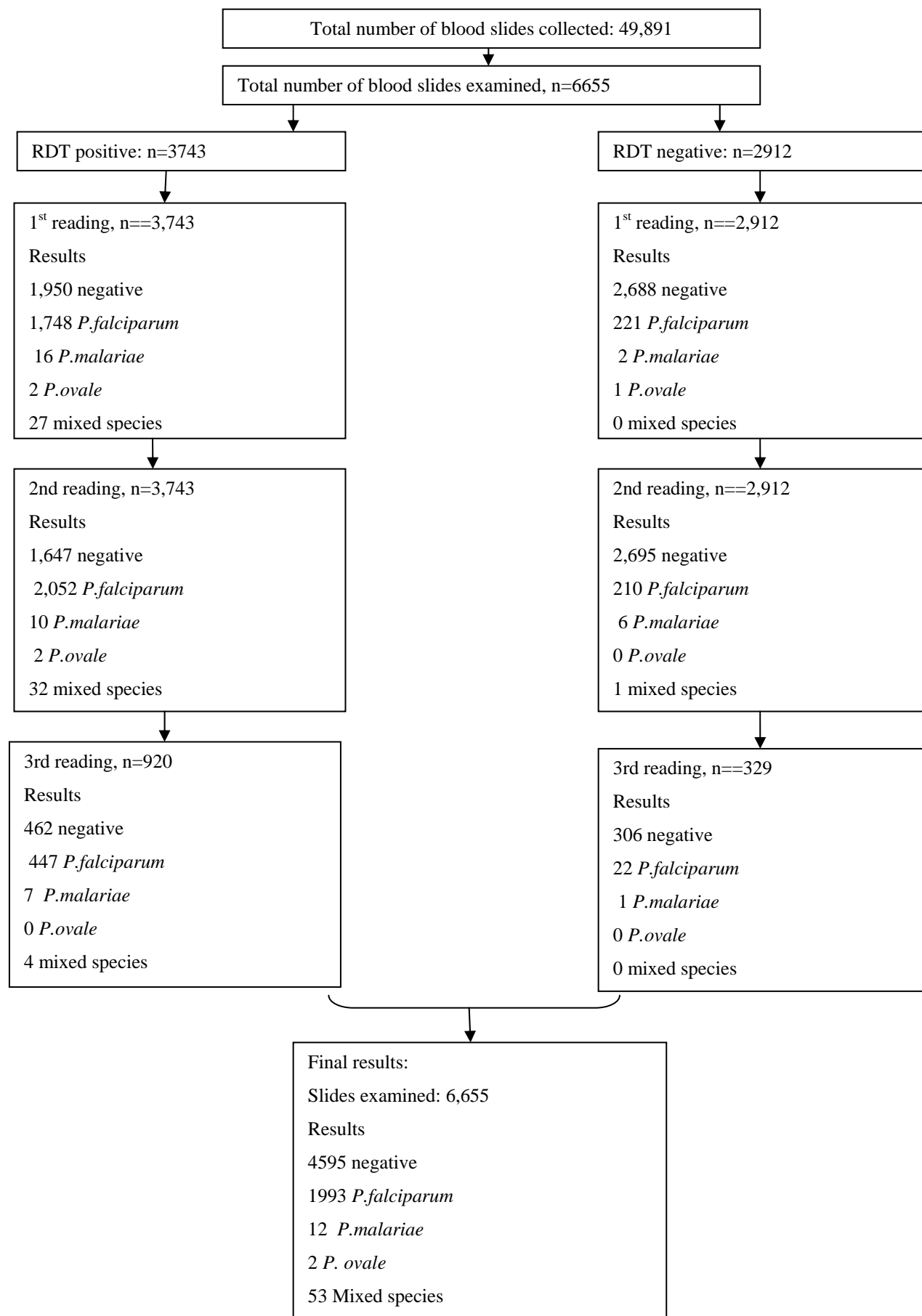

Supplement: Additional file 2 — Microscopy results flowchart. A flow chart showing the numbers of slides examined and the microscopy results, including discrepant results, in the school malaria surveys in Kenya, 2008-2010. [file 1475-2875-9-306-S2.PDF]
